# Identification and Characterization of a Predominant Hydrophobin in the Edible Mushroom *Grifola frondosa*

**DOI:** 10.3390/jof10010025

**Published:** 2023-12-29

**Authors:** Bo Song, Wenjun Wang, Chunhui Jia, Zhiqiang Han, Jiyuan Yang, Jiuxia Yang, Zhenzhou Wu, Haijin Xu, Mingqiang Qiao

**Affiliations:** 1 The Key Laboratory of Molecular Microbiology and Technology, Ministry of Education, College of Life Sciences, Nankai University, Tianjin 300110, China; 15028259109@163.com (B.S.);; 2 School of Life Science, Shanxi University, Taiyuan 030000, China

**Keywords:** maitake, hydrophobins, transcription levels, life cycle, fungal development

## Abstract

Hydrophobins (HFBs) are a group of small, secreted amphipathic proteins of fungi with multiple physiological functions and potential commercial applications. In this study, HFB genes of the edible mushroom, *Grifola frondosa*, were systematically identified and characterized, and their transcriptional profiles during fungal development were determined. In total, 19 typical class I HFB genes were discovered and bioinformatically analyzed. Gene expression profile examination showed that *Gf.hyd9954* was particularly highly upregulated during primordia formation, suggesting its major role as the predominant HFB in the lifecycle of *G. frondosa*. The wettability alteration profile and the surface modification ability of recombinant rGf.hyd9954 were greater than for the *Grifola* HFB HGFII-his. rGf.hyd9954 was also demonstrated to form the typical class I HFB characteristic-rodlet bundles. In addition, rGf.hyd9954 was shown to possess nanoparticle characteristics and emulsification activities. This research sheds light on the regulation of fungal development and its association with the expression of HFB genes.

## 1. Introduction

Hydrophobins (HFBs) are surface-active proteins that have the ability to form robust and well-organized surface layers with amphipathic behavior at air/water or air/solid interfaces, and they are extensively found in filamentous fungi [1]. HFBs consist of 80–170 amino acids, although they can be more than 400 amino acids in length when the classification includes polyhydrophobins [2], and all share the common feature of eight cysteine residues with sequence homology within and across species [3,4,5]. HFBs comprise four loops formed by disulfide bonds between the eight conserved cysteine residues. These loops within the molecule result in hydrophilic and hydrophobic regions, each playing a crucial role in properties associated with self-assembly or attachment to surfaces [6].

In addition to the eight unifying Cys residues, HFBs are also characterized by the specific spacing patterns of the primary sequences. The eight consensus Cys residues show the following spacing pattern: C-X_6–7_-CC-X_33–41_-C-X_19–25_-C-X_5_-CC-X_12–17_-C in class I HFBs, which differs from the consensus Cys spacing pattern of class II HFBs, namely C-X_9–10_-CC-X_11_-C-X_16_-C-X_8–9_-CC-X_10_-C; the numbers indicate the numbers of amino acids occurring between Cys residues, consecutively numbered from the N to the C terminus [7,8]. Furthermore, class I and class II HFBs can be distinguished based on hydrophobicity patterns and solubility properties. Class I HFBs enable self-assembly into rodlets similar to amyloid fibrils that can be solubilized only in strong acids, whereas class II HFBs form less regular and stable elastic monolayers soluble in solvents, which can be dissociated under relatively mild conditions [9]. Due to their amphipathic nature and the superstructure of the assembly, as functional entities, hydrophobins have been found to play important roles in various fungi [10]. All HFBs are able to self-assemble into suprastructural organization, with highly ordered amphipathic layers occurring in the conidia or aerial hyphal surfaces that assist the filamentous fungi to fulfill life activities and various fungal processes, such as hyphal growth [11], primordium differentiation [12,13], and fruiting body development [12,14,15]. These proteins are also associated with sporulation and sexual development [5,16,17,18], as well as the capacity to become pathogenic [19,20,21,22].

HFBs have been reported to act as high-profile natural nano-bioactive materials to be used in biosensors [23,24,25], enzyme immobilization [26], and emulsification [27]. Other applications have been explored in drug nanosuspensions, drug delivery [28,29] and tissue engineering [30,31]. Recently, the applications of HFBs were broadened to include the modification of the cryo-electron microscopy support film [32,33]. Being an industrially important protein, identification and heterologous expression of new HFB molecules are urgently needed.

HFBs are a group of secretory proteins in fungi, with varying family members among different fungal species [34]. Thus, investigating the HFB families of fungi should shed light on how HFBs affect fungal development and could broaden their industrial applications. For example, around 74 putative HFB genes have been identified in eight species of *Aspergillus* [35], with six putative HFB genes reported in *Aspergillus nidulans* [36,37], and three in *Bionectria* (*Clonostachys*) *rosea* [38]. Recently, researchers have found that *Pleurotus ostreatus* contains 40 putative HFB genes [39], the white rot fungus *Coriolopsis trogii* contains 36 HFB genes [18], and the endophytic fungus *Clonostachys solani* has three HFB genes [22].

Among the fungal HFB families, the number of HFB members per species was variable, as was their expression pattern during the developmental phases of the fungus [40]. We postulated that the HFB genes, which have significant medicinal and dietary value, exhibit distinct expression profiles in the edible fungus Grifola frondosa. Recently, cDNA sequencing of G. frondosa led to the discovery of a new HFB-encoding gene, *hgfII* (GenBank: QTF98737.1, HGFII) [41]. Its efficacy and applications were subsequently investigated [42]. To further explore the HFB genes in *G. frondosa*, this study conducted whole-genome sequencing and determined the HFB gene expression patterns across fungal developmental stages. This resulted in the identification of a putative key HFB in *G. frondosa*, which exhibited significantly high transcript levels and predominant profiles throughout its life cycle.

## 2. Materials and Methods

### 2.1. Fungal Strains and Culture

The *G. frondosa* CICC^®^50075 strain used in this study was maintained in our laboratory on potato dextrose agar (PDA) (potato 200 g/L, glucose 20 g/L, KH_2_PO_4_ 3.0 g/L, MgSO_4_·7H_2_O 1.5 g/L, and agar 20 g/L, pH 6.0) solid slants, with subculturing every three months. The mycelium of *G. frondosa* was transferred from the PDA slants onto PDA Petri dishes (90 mm diameter) and darkly cultured at 26 °C until the colony covered the entire surface of the agar, to serve as starter plates for use in culture experiments. To culture the fungus, a 15-day-old solid culture of *G. frondosa* mycelia on PDA was harvested into a 4-mL centrifuge tube containing 3 mL sterile ddH_2_O and three 2.0 mm-diameter stainless steel beads, and the mycelium was subsequently homogenized with a blender (SCIENTZ-48^®^; Scientz Biotechnology, Ningbo, China) at 60 Hz for 2 min. An aliquot (20 mL) of the mycelial homogenate was injected into each sterilized fungal culture jar (containing chestnut sawdust 45%, corncob 10%, cottonseed hull 18%, corn flour 5%, wheat bran 20%, gypsum powder 1%, and sucrose 1%). To maintain humidity, several drops of deionized water were put into the autoclaved medium bags containing the PDB liquid medium and sealed with sterilized spongy stoppers before placing the bag culture into the incubator with RH 75–85% at 25 °C in the dark. When the primordia-forming stage was reached, the culture conditions were changed to 18 °C, high (80%) humidity, and 12-h light/12-h dark cycles [43]. The reagents used in the PDA and PDB media were purchased from Sangon Biotech (Shanghai, China) and the fungal culture medium were kindly donated by SIJI FUNGI^®^ (Luzhou, China).

### 2.2. High-Throughput Genome Sequencing and De Novo Assembly

HFB-encoding genes can be identified using computer-aided bioinformatics, but accurate datasets from fungal genome projects are required. By recognizing patterns of similarity between conserved residues, algorithms can be used to predict the translation of hypothetical genes into proteins [35]. The ~6 g of 15-day cultured inoculum mycelia on PDA Petri dishes of *G. frondosa* was harvested, the whole genome of the specimens was extracted, quality control was conducted, and the genome was sequenced using the Sanger/Illumina 1.9 method on the Illumina^TM^ HiSeq PE150 platform by the Beijing Allwegene Technology Co., Ltd. (Beijing, China). The sequencing data of each strain after quality control were assembled using the SPAdes (v3.13.0) software to obtain sequence files that reflect the basic information of the sample genome. The assembly quality was statistically analyzed, and scaffold sequences greater than 200 bp were selected for subsequent analysis. Further details are provided in the Supplementary Materials.

### 2.3. Identification of Putative Hydrophobins

The prediction of fungal genomes was performed with de novo Augustus methods. We filtered the *G. frondosa* CICC^®^50075 sequenced genome for putative HFBs, for which the amino-acid (aa) sequences were determined. Putative HFBs were confirmed using several criteria, namely protein length (80 to 400 aa), the presence of eight conserved cysteine residues, and the presence of domains matching the Pfam (PF01185/IPR001338) pattern to meet the HFB criteria and verify their function as HFBs [44].

### 2.4. Bioinformatic Analysis of Hydrophobins

Bioinformatic predictions of the number of amino acids, molecular weight, theoretical pI, composition of charged residues, and Grand Average of Hydropathicity (GRAVY) were performed on ProtParam (https://web.expasy.org/protparam/, acccessed on 28 August 2022) [45]. The predicted signal sequences were manually removed from the amino-acid sequences of the candidate HFBs and their secretion abilities were evaluated on SignalP 5.0 (https://services.healthtech.dtu.dk/service.php?SignalP-5.0, acccessed on 2 September 2022), while the subcellular protein localizations of candidate HFBs, were predicted on the WoLF PSORT (https://wolfpsort.hgc.jp/, acccessed on 15 September 2022) bioinformatics tool. The neighbor-joining (NJ) (1000 bootstrap replicates) method on MEGA 7.0 software [46] was used to build phylogenetic trees of the HFBs from *G. frondosa* strains CICC^®^50075 and 9006-11 (submitted GenBank assembly: GCA_001683735.1). The tertiary structures of target hydrophobins were predicted on the SWISS-MODEL website (https://swissmodel.expasy.org/, acccessed on 18 September 2023).

### 2.5. RNA Extraction and cDNA Synthesis

The fungal mycelia, primordia, and young and mature fruiting bodies of *G. frondosa* CICC^®^50075 were incubated in our lab as aforementioned. The forementioned specimens were frozen and homogenized to a fine powder in liquid nitrogen using disposable pyrogen-free and RNase-free EP tubes and pestles. Then the total RNA was extracted using the RNAprep Pure Plant Kit (Tiangen, Beijing, China) and diluted 10 times with Rnase-free water for the cDNA synthesis according to the manufacturer’s instructions. The cDNA was synthesized with the PrimeScript^®^ RT reagent kit with gDNA Eraser (Takara, Dalian, China) via a Bio-Rad PCR machine. The reverse transcription quantitative PCR (qRT-PCR) reaction was performed using the 2 × SYBR Green qPCR Master Mix (Bimake, Shanghai, China), following the manufacturer’s instructions. RNA and cDNA were measured and validated using a NanoDrop 2000 ultramicro-spectrophotomer (Thermo Fisher Scientific, Waltham, MA, USA).

### 2.6. Quantification of Hydrophobin Gene Transcription

HFBs have been isolated from many species of fungi, and their expressions were found to be developmentally regulated [4]. To investigate the expression pattern of HFBs in *G. frondosa*, expression trends were assessed by qRT-PCR. Four time points during fungal development were chosen for this study, namely mycelia germination, formation of the primordia, young fruiting body, and mature fruiting body. Mycelia were cultured in continuous darkness at 25 °C for 15 d on PDA plates. Subsequently, mycelia of *G. frondosa* CICC^®^50075 were inoculated into 300 mL fungal substrate culture bags and cultured in the dark (by covering the bags with aluminum foil) at 25 °C at RH 75–85% for one month. Primordia differentiated after the mycelia filled the substrate and began to tangle up, forming mycelial knots, and then developed into fruiting bodies under natural light, 85% humidity, and a low temperature (16–18 °C). Total RNA of the aforementioned samples was extracted with the RNAprep Pure Plant kit (Tiangen, Beijing, China), followed by reverse transcription with the PrimeScript™ RT reagent kit with gDNA Eraser (Takara, Dalian, China). Then, each of the 2 μg of RNA was used for quantitative real-time PCR (qPCR) analysis with a three-step method [39], using the real-time fluorescence quantitative PCR cycler 2 × SYBR Green qPCR Master Mix (Bimake, Shanghai, China) on an ABI StepOnePlus Real-Time PCR System (Applied Biosystems, CA, USA) with paired primers (Table S3). The glyceraldehyde-3-phosphate dehydrogenase gene, *gapdh*, from *G. frondosa* CICC^®^50075 was used as an internal housekeeping standard to normalize expression levels (the sequence of *gapdh* was listed in Supplementary Materials). Each qRT-PCR reaction was carried out in three independent biological replicate assays, and data were presented as the mean values of the three replicates with the corresponding standard deviations. The relative expression level of hydrophobin genes among mycelia (Myc), primordia (Pri), young fruiting body (Yfb) and mature fruiting body (Mfb) were analyzed by qRT-PCR and the raw data were calculated by the 2^−ΔΔCt^ method [41,47] and then normalized to those of the mycelia cultured on PDA. qRT-PCRs were carried out in triplicate with each of the different cDNA samples and relative transcript level was normalized to the corresponding expression level of *gapdh*, then analyzed by one-way analysis of variance (ANOVA) using Prism version 8.4 (GraphPad Software, Inc., La Jolla, CA, USA). Data were expressed as means ± standard deviations (SD).

### 2.7. Heterologous Expression of Hydrophobin in Pichia Pastoris (Komagataella phaffii)

To achieve efficient purification and better solubility of the recombinant hydrophobin, the 6*His-tag was fused to the C terminus of the mature Gf.hyd9954 amino-acid sequence. Codon optimization of the *Gf.hyd9954*, primer synthesis, and DNA sequencing and the recombinant *Escherichia coli* strain were genetically engineered by GENEWIZ (Suzhou, China). The schematic diagram of plasmid constructs is displayed in Figure S1a. The recombinant HFB rGf.hyd9954 was expressed and purified using the standard protocols (see the Supplementary Materials) for a complete description [48]. Restriction enzymes were obtained from Takara (Dalian, China). Polymerase chain reaction (PCR) products were purified using the MiniBEST Agarose Gel DNA Extraction Kit^®^ (Takara Bio Inc., Shiga, Japan). Plasmids were extracted using the TIANprep Mini Plasmid Kit^®^ (Tiangen, Beijing, China). Prestained protein standards (#M221) were supplied by Genstar (Beijing, China). The High-Affinity Ni-Charged Resin FF was supplied by GenScript^®^ (Nanjing, China). The ECL kit was from SparkJade^®^ (Jinan, Shandong, China), and the anti-His mAb was from GenScript (Nanjing, China). The recombinant *E. coli* strain was cultured in Luria broth (LB) medium (yeast extract 5 g/L, tryptone 10 g/L, NaCl 10 g/L) at 37 °C for 8 h, while the yeast *(P. pichia*) strain was cultured in buffered minimal glycerol medium [BMG, 100 mM KH_2_PO_4_ (pH 6), 1.34% yeast nitrogen base with (NH_4_)_2_SO_4_ and without amino acids, 4 × 10^−5^% biotin, and 1.0% glycerol] and fermented in buffered minimal medium [BMM, 100 mM KH_2_PO_4_ (pH 6), 1.34% yeast nitrogen base with (NH_4_)_2_SO_4_ and without amino acids, 4 × 10^−5^% biotin, and 1% methanol] (for shake flask fermentation) with 220 rpm at 28 °C for 96 h. The *E. coli* recombinant strain was used to propagate the recombinant plasmid, while the *K. pastoris* strain GS115 was used for the heterologous expression of the HFB gene *Gf.hyd9954*. 

### 2.8. Water Contact Angle (WCA) Determination

Water contact angle determination was carried out to evaluate the surface modification profile of the purified rGf.hyd9954 [49]. Teflon film and mica slices were utilized as hydrophobic and hydrophilic surfaces, respectively. One 50 μL drop of HFB solution (100 μg/mL) was incubated on freshly cleaved Teflon film and mica slices overnight at room temperature before removing the surplus and rinsing the slices with ddH_2_O. A hot (~60 °C) 2% sodium dodecyl sulfate (SDS) solution was used to rinse the coated surface to evaluate the resilience profile of the rGf.hyd9954 self-assembly coating layer. WCA determination was performed with a KSV Contact Angle Measurement System (KSV Instruments Ltd., Espo, Finland). A volume of 5 μL ddH_2_O was dropped onto the dried HFB-coated surfaces [50]. Each sample was measured in three different regions, and the results were presented as the mean ± SD.

### 2.9. X-ray Photoelectron Spectroscopy (XPS) Analysis

Due to the remarkable self-assembly characteristics of HFBs, they are able to modify the interface by forming an intact elastic film. XPS was carried out to determine the elemental composition of the silicified glass surfaces with or without the rGf.hyd9954 coating. XPS were measured using an Axis Ultra DLD spectrometer (Kratos Analytical Ltd., Manchester, UK), employing a monochromated Al-Ka X-ray source (hV = 1486.6 eV) with hybrid (magnetic/electrostatic) optics and a multi-channel plate and delay-line detector. A concentration of 100 μg/mL HFB solution was spread on a 0.5 cm × 0.5 cm silicified glass slide and left overnight at room temperature. The next day, the remaining solution was removed, followed by rinsing three times with ddH_2_O, and finally drying with nitrogen gas. Spectra of C, N, O, and Si were recorded. Data files were processed using CasaXPS^®^ software (v. 2.3.18PR1.0, Casa Software Ltd., Teignmouth, UK), and the reported XPS energies are the binding energies expressed in eV [24].

### 2.10. Thioflavin T (ThT) Fluorescence Measurement

Thioflavin T (ThT) is a benzothiazole dye that acts as a “molecular rotor” and exhibits enhanced binding to amyloid fibrils, resulting in it being commonly used to detect amyloid fibrils. The change in thioflavin T (ThT) fluorescence was performed to monitor fibril formation from rGf.hyd9954. The aqueous solution exhibited a prominent peak at 482 nm. The interaction between the ThT and rGf.hyd9954 solutions was assessed using a multimode plate reader (Perkin Elmer, Akron, OH, USA) before and after vortexing. In this experiment, a solution containing 50 μg/mL rGf.hyd9954 and a stock solution of 1.5 mM ThT were prepared in ddH_2_O. Subsequently, 400 μL of the rGf.hyd9954 solution was transferred to a 1.5 mL microcentrifuge tube, and 4 μL of the ThT stock solution was added for measurement purposes. The spectrum was then measured with an excitation wavelength of 435 nm, while the scanning wavelength ranged from 450 to 600 nm at 10 nm intervals. A blank ThT solution was employed as a control in this experiment [51].

### 2.11. Atomic Force Microscopy (AFM) Detection

As amphipathic biopolymers, HFBs are capable of forming elastic layers on hydrophobic/hydrophilic surfaces. In this study, 20 μL of 20 μg/mL rGf.hyd9954 was dropped onto the mica slices and allowed to sit for 5 min, followed by the removal of excess liquid. The droplets were subsequently rinsed three times with ddH_2_O and dried using nitrogen gas. The surface topography was visualized using a Bruker Dimension Atomic Force Microscope (Bruker, Eltlingen, Germany). The scan area was configured with a scanning frequency of 1.5 Hz and a resolution of 512 points/line. The image processing procedure involved flattening the captured images to eliminate any potential tilt with NanoScope Analysis (version 1.5, Bruker AXS Corporation, Madison, WI, USA) [52]. Furthermore, in the present study, we examined the homology of the putative 3D structure of rGf.hyd9954 with that of a class I HFB, 5w0y.1.A, found in *Serpula lacrymans*.

### 2.12. Determination of Surface Hydrophobicity with 1-Anilino-8-naphthalenesulfonate (1,8-ANS)

The HFB surface hydrophobicity was determined by using 1,8-ANS as the hydrophobic fluorescent probe. rGf.hyd9954 lyophilized powder was dissolved in ddH_2_O at a concentration of 100 μg/mL by ultrasonic treatment for 5 min at room temperature, and 2.57 mg of 1,8-ANS powder (purchased from TCI Chemicals, TCI Europe N.V., Zwijndrecht, Belgium) was dissolved in 1 mL ddH_2_O. An aliquot (500 μL) of the HFB solution was transferred to a new 1.5 mL centrifuge tube, to which were added 1.25 μL of 1,8-ANS stock solution. After thorough mixing, the mixture was transferred into a microplate for detection. Fluorescence intensity (FI) was measured at continuous spectrum scanning between the wavelengths of 390 nm and 700 nm with 10 nm slit widths using a microplate reader (Enspire, PerkinElmer, Waltham, MA, USA). An aliquot (500 μL) of 1,8-ANS (20 mM) or rGf.hyd9954 aqueous solution acted as the control [53].

### 2.13. Particle Size and Zeta Potential Measurements

Dynamic light scattering (DLS) analysis was performed at 25 °C. The lyophilized powder of rGf.hyd9954 was dissolved in 1 mL of ddH_2_O at a concentration of 100 μg/mL with ddH_2_O. Zeta potential was employed to determine the charge on the surface of particles of rGf.hyd9954 using a Zeta Sizer 5000 instrument (Malvern Instruments, Herrenberg, Germany). Particle size and zeta potential measurements were carried out on three replicates, and the results were expressed as mean ± SD [54].

### 2.14. Emulsification Assay

An emulsification assay was conducted to evaluate the surfactant capacity of rGf.hyd9954. Soybean oil was used as the dispersion solvent, and the HFBs served as emulsifiers. A 1.5 mL aliquot of ddH_2_O or HFB aqueous solution (100 μg/mL) was added to 2-mL test tubes. Then, 6% of 2 mL (*v*/*v*) soybean oil was added to each test tube to form the emulsification system. Homogenization was achieved by vortexing vigorously for 2 min, followed by ultrasonication at room temperature for 20 min, followed by allowing to rest for 72 h. Finally, they were visually checked by microscopy for phase clarity and emulsification stability [41].

### 2.15. Statistical Analysis

The data were presented as the mean ± standard deviation (SD). Statistical analysis in this study was conducted using Microsoft Excel (Microsoft Office 2020, Microsoft Corp., Redmond, WA, USA) or GraphPad Prism 8.4 (GraphPad Software, Inc., La Jolla, CA, USA) software. A student’s *t*-test was employed to compare two groups, whereas one-way analysis of variance (one-way ANOVA) was utilized for comparisons involving more than two groups. Statistical significance was determined at a *p*-value of 0.05 or less.

## 3. Results and Discussion

### 3.1. Sequence Search and Annotation of Putative Hydrophobin Genes in Grifola frondosa

**Figure 1:**
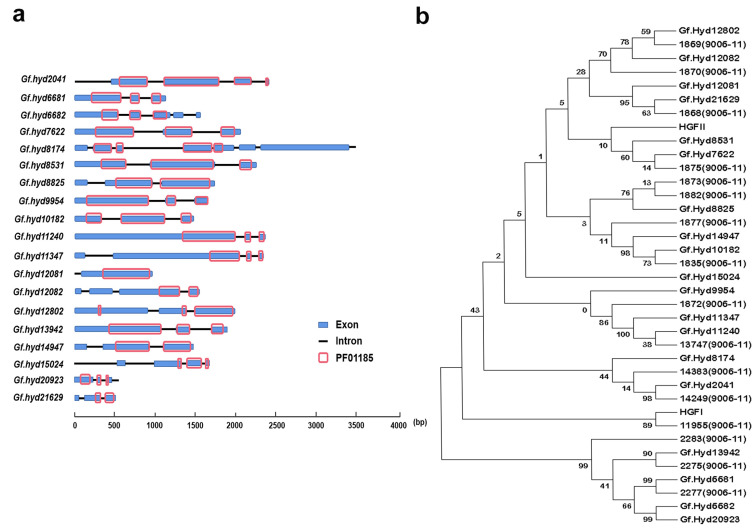
The primary gene structure and phylogeny analysis of hydrophobin genes among *G. frondosa*. (**a**) Gene structure of the hydrophobin genes. PF01185 is the domain of “fungal hydrophobin”. (**b**) Phylogeny between two strains and their putative hydrophobin genes. *G. frondosa* CICC^®^50075 and 9006-11 are the two sub-lineages of *G. frondosa.* In order to analyze the hydrophobin genes of these two strains and their own phylogeny, the Neighbor-Joining Tree (1000 bootstrap replicates) was conducted via MEGA7 software. Putative hydrophobin genes *Gf.hydXXXX* are from CICC^®^50075, while the others are from lineage 9006-11.

HFBs exhibit diversity in most Basidiomycete fungal species, corresponding to their pleiotropic functions. In the current study, we performed whole-genome sequencing and cross-checked the sequence patterns of the proteins encoded according to Pfam 01185 of the unigenes in the *G. frondosa* genome. Twenty-two candidate HFBs were first identified in this study. Notably, three putative HFBs only contained seven (Gf.hyd15802 and Gf.hyd19717) or six (Gf.hyd4500) Cys residues in the amino-acid sequences. Several studies have suggested that some HFBs contain only six, seven, or even nine Cys residues as opposed to the canonical eight [38,55,56,57]. According to the amino-acid sequences of the remaining 19 confirmed typical 8-Cys-residue-hydrophobins, the order of the eight Cys residues in the *G. frondosa* hydrophobin primary structures forms a characteristic pattern: C-X_7/8_-CC-X_13/29/31/32_-C_12/13_-C-X_5/6_-CC-X_11/12_-C (C represents cysteine, X represents other amino acids except cysteine and tryptophan). Subsequently, phylogenetic analysis was conducted using MEGA7.0 to describe the phylogeny between the HFBs from two lineages of different *G. frondosa* strains. The phylogenetic tree contains several clusters with sub-clades of the putative HFBs from the two strains of *G. frondosa* (CICC^®^50075 and 9006-11), indicating that there is a close (perhaps cognate) relationship between the HFBs of the two strains, with each sub-clade showing a level of homology; on the contrary, the HFBs of each sub-clade displayed a polymorphic distribution (Figure 1). The basic information about the *G. frondosa* genome is shown in the Supplementary Materials.

### 3.2. Bioinformatic Analysis of Hydrophobins

In this study, 19 novel HFBs were identified for the first time, with the majority of them ranging from 100 to 142 amino acids and containing eight conserved cysteine residues. However, Gf.hyd12082, Gf.hyd11347, and Gf.hyd11240 each had relative redundancy at the N-terminus before the first cysteine residue, resulting in sequences of 232, 376, and 392 amino acids, respectively, along with the conserved eight cysteine residues and spacing patterns; the molecular weight of these HFBs was between 9723.19 and 36,493.27 Da. Nevertheless, they still fit the characteristics of class I HFBs. Zhang et al. (2022) reported PCST1, a large HFB from *Arabidopsis thaliana*, with 403 amino acids and a molecular weight of 45,323.60 Da [58]. The primary sequence structures of the genes encoding these hydrophobic proteins mostly contain three to five exons, although *Gf. hyd12081* has one exon and *Gf. hyd8174* has seven exons. These results indicate that, although they belong to the same HFB family, their sequence structures are polymorphic (Table 1).

Furthermore, out of the 19 novel HFBs, Gf.hyd2041 was predicted to be located in the cytoplasm, whereas the others were located extracellularly. Nevertheless, there were no disparities between Gf.hyd2041 and other HFBs (except for the none match items for the Gf.hyd6682, Gf.hyd12081, and Gf.hyd20923), all of which were enriched with respect to nine Gene Ontology (GO) terms: three molecular function terms (structural constituent of cell wall, molecular function, and structural molecular activity) and six cellular component terms (external encapsulating structure, cellular anatomical entity, cell wall, cellular component, fungal type cell wall, and cell periphery) but no biological process terms. From analysis of their physicochemical properties, the HFBs can be acidic, neutral, or alkaline proteins (with pI values ranging from 3.57 to 8.50), but they are all hydrophobic proteins (with a GRAVY index ranging from 0.270 to 0.951). Except for Gf.hyd12082 and Gf.hyd21629, the other 17 HFBs contain a signal peptide for an improved exocytosis profile (Table 2). The hydropathicity of the proteins was determined using the Kyte and Doolittle scale [59].

### 3.3. Expression Profile Analysis of Hydrophobin Genes during the Life Cycle of G. frondosa CICC^®^50075

During the life cycle of fungi, numerous genes are expressed synergistically, and their transcript levels are continuously adjusted to suit the needs of mushroom development. It has been reported that HFB genes are expressed temporally across the life cycle of mushrooms [60]. Some research also reported that one specific HFB gene was significantly expressed at each time point, like the primordia stage. Kim et al. (2016) found that the *FBH1* gene affects the growth rate and primordia formation of *Pleurotus ostreatus* [61]. For its part, in *Flammulina velutipes*, the *Hyd9F* is highly expressed in the primordia and is involved in the formation of aerial hypha knots [13].

**Figure 2:**
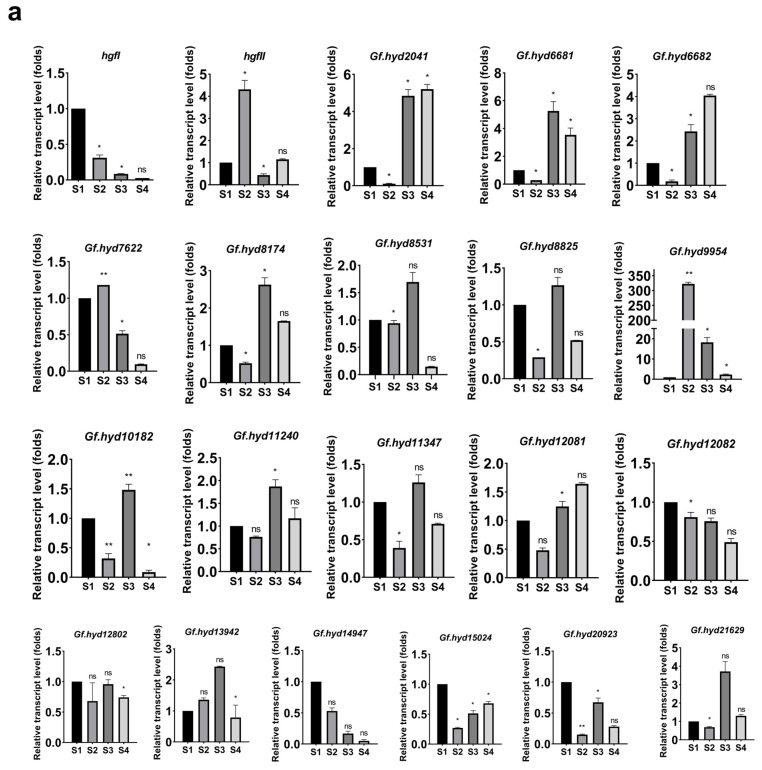

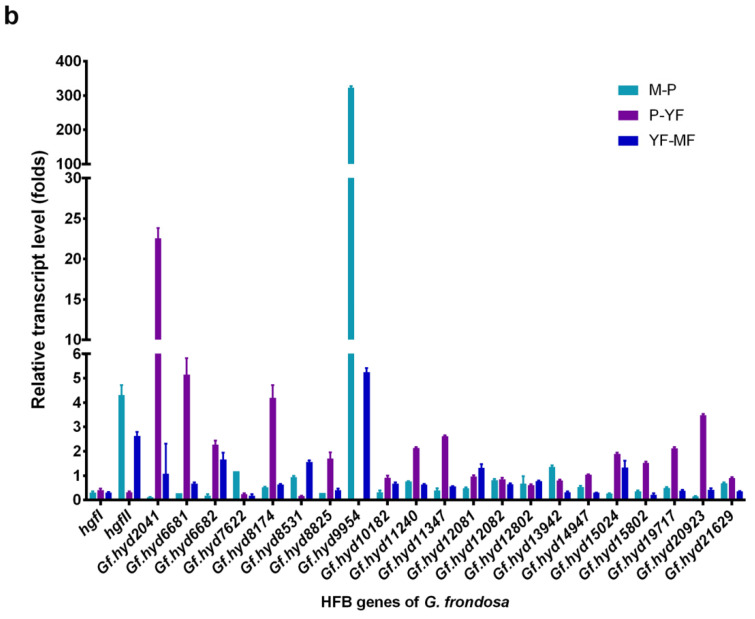
The expression pattern of hydrophobin genes during one-life cycle of *G. frondosa* CICC^®^50075. (**a**) The relative expression levels of typical hydrophobin genes at the mycelia, primordial stage, and fruiting body development stages; all the expression levels were calibrated with the levels of the mycelia stage. S1, the mycelia (Myc) stage; S2, the primordial stage (Pri); S3, the young fruiting body (Yfb) stage; S4 represents the mature fruiting body (Mfb) stage. (**b**) The relative expression levels of hydrophobin genes that compared to former stage. * represents *p* < 0.05, ** represents *p* < 0.01, ns represents nonsignificant difference.

The current research reported that the *G. frondosa* CICC^®^50075 transcripts of many novel HFB genes were detected in abundance at various time points of fungal development. Expression of genes *hgfI*, *Gf.hyd15024*, and *Gf.hyd20923* was significantly upregulated (*p* < 0.05) at the germination stage, compared with the primordia and young fruiting body stages (Figure 2a), and might be the critical genes for germination. Because *hgfII* and *rGf.hyd9954* were abundantly expressed at the primordial stage, it is assumed that they could be the predominantly expressed HPB genes. Moreover, transcript levels of seven HFB genes (*Gf.hyd2041*, *Gf.hyd6681*, *Gf.hyd6682*, *Gf.hyd8174*, *Gf.hyd11240*, *Gf.hyd11347*, and *Gf.hyd20923*) were significantly abundant, which might be related to the differentiation of the fruiting body. In contrast, expression of the *rGf.hyd9954* gene was particularly attenuated during the transition phase from the primordium to the young fruiting (Yfb) body stage (Figure 2a). A remarkably high transcript level of *rGf.hyd9954* was observed at the primordia stage of *G. frondosa* strain CICC^®^50075, which was about 380-fold higher than that at the earlier germination stage. Intriguingly, not only in the primordia stage, *rGf.hyd9954* was also liberally expressed at the mature fruiting (Mfb) development stage. Xu et al. (2021) found that in *Pleurotus ostreatus*, *Vmh3* represented over 90% of the HFB gene expression, as it is predominantly expressed at almost every stage of the life cycle, excluding the mature fruiting body stage [39], while the *fv-hyd1* gene was notably transcribed at the primordia stage of *Flammulina filiformis* [62]. The mature fruiting body stage may have a limited requirement for HFBs, reflected in the attenuation of expression levels of ten HFB genes, while the expression of only two HFB genes (*hgfII* and *rGf.hyd9954*) was upregulated at this stage. Regarding the Yfb-to-Mfb transition stage, the highly expressed genes were *hgfII*, *rGf.hyd9954*, *Gf.hyd12802*, and *Gf.hyd21629*. Taking these results together, we inferred that *rGf.hyd9954* might be the main HFB gene responsible for triggering fruiting in *G. frondosa* CICC^®^50075.

HFB genes display temporally expressed patterns, reflecting their importance in inducing fungal development. The results of the study suggest that the expression of an HFB gene can vary in its regulation at different stages of fungal development. Based on these results, it was discovered that the HFB genes were expressed in a coordinated and time-regulated manner, aligning with the developmental necessities throughout the life cycle of *G. frondosa*. For example, the *Gf.hyd9954* gene encodes a novel HFB deeply involved in the fruiting process, which is specifically expressed at the primordia and fruiting body stages (Figure 2b). Furthermore, to easily obtain the rGf.hyd9954 and reveal the inherent mechanisms of the rGf.hyd9954 exhibiting predominant expression levels, the heterologous expression and purification of recombinant rGf.hyd9954 in *Pichia Pastoris* GS115 were successfully performed. (Figures S1 and S2), and the following characterization studies were performed.

### 3.4. rGf.hyd9954 Exhibits Wettability Alteration Ability Confirmed by Water Contact Angle

HFBs are highly surface-active proteins and are able to exhibit wettability by self-assembly into an amphipathic film at the interface between two phases [7]. In our research, the amphipathic nature of the proteins was evaluated by the water contact angle (WCA) with the Teflon surface (hydrophobic) and mica surface (hydrophilic). After coating with rGf.hyd9954, WCA was 31.09% reduced on the Teflon surface, a result that was similar to that of the HGFII-his-coated Teflon surface (32.34%) (Figure S4), compared with an 133.97% increase in WCA on the mica slice, which was significantly higher than that of the HGFII-his coating mica surface (96.16%). The results indicate that rGf.hyd9954 was able to convert the wettability of the coated surface in the same way as natural HFBs. Several studies have indicated that some HFBs changed the WCA on hydrophobic and hydrophilic surfaces by 40–60% [63,64,65]. In addition, Lo et al. (2014) found that Class I HFB layers formed on a graphite surface were resistant to alcohol, acid, and basic washes [52]. To evaluate the stability of the self-assembly film of rGf.hyd9954, a hot 2% SDS solution was applied to the rGf.hyd9954-coated surfaces, with ddH_2_O-rinsed coated surfaces being used as the control. The confirmed class I hydrophobin HGFII-his-coated surfaces under the same treatment served as the control [42]. The hot 2% SDS solution indeed reduced the wettability of the coated surfaces; the hydrophobicity of the rGf.hyd9954-coated Teflon surface was attenuated from 59.48 ± 2.47° to 62.17 ± 4.15°, whereas the angle of coating of the mica sheet decreased from 20.73 ± 0.98° to 17.43 ± 2.19°. The amplitude of the attenuated WCA on the coated Teflon surfaces was about 4.52% after the hot 2% SDS solution was rinsed onto the surfaces, which was significantly different from the 22.93% of the HGFII-his-coated surfaces. As for the WCA on mica sheets, after being rinsed by hot 2% SDS solution, the WCA changed by 15.91% on the rGf.hyd9954-coated surface and by 58.46% on the HGFII-his-coated sheets (Table 3 and Figure S4g,i). As a consequence, rGf.hyd9954 exhibited the attractive properties of wettability alteration profiles and the outstanding stability of the self-assembled film.

### 3.5. The Modification Ability of *rGf.hyd9954* on a Silicon Slice

**Figure 3:**
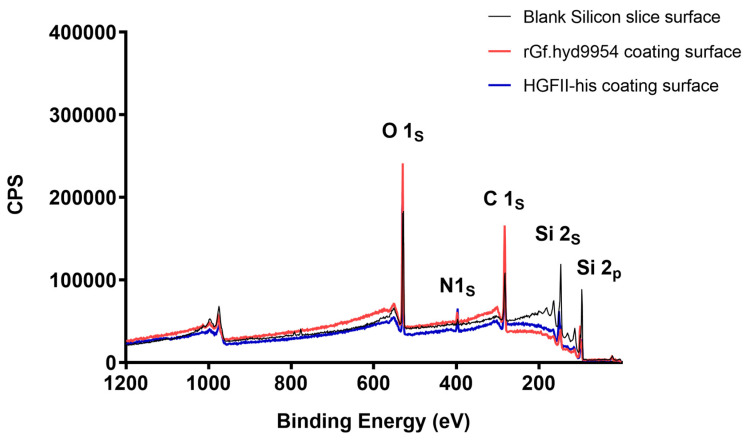
General XPS spectrum of blank silicon slice surfaces and hydrophobin-containing silicon slice surfaces. Hydrophobin-containing silicon slice surfaces XPS spectra of rGf.hyd9954 and HGFII-his.

X-ray photoelectron spectroscopy technology is an advanced technique commonly used for the accurate measurements of inner-atomic electron binding energy and electronic transition and is therefore used to analyze the elemental composition and content of various material surfaces [24,66]. Class I HFBs can self-assemble into a dense protein layer on the surfaces. Therefore, we used XPS to analyze the elemental distribution of the silicon slice surface after modification with rGf.hyd9954. As shown in Figure 3, compared with the blank silicon slice, the silicon slice modified with rGf.hyd9954 showed that the counts per second (CPS) of the N1s peak increased to about 22.6%. The CPS of Si2s decreased by 48.7%, and Si2p attenuated by 59.4%. These results indicate that rGf.hyd9954 indeed possesses self-assembly properties and successfully assembles into a protein film on the hydrophobic silicon slice surface.

### 3.6. Monitoring of Self-Assembly by the Thioflavin T- (ThT-) Binding Assay

**Figure 4:**
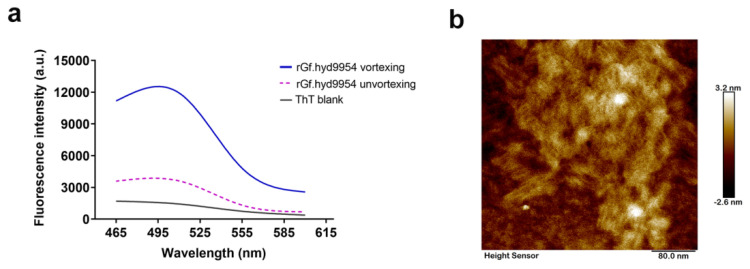
The characterization of the rGf.hyd9954 assembly. (**a**) The ThT analysis of the *rGf.hyd9954*. ThT is a fluorescent dye that is commonly used for monitoring the amyloid formation by proteins and peptides. The hydrophobin assembly frequently accompanied by the formation of the cross-β structure. The emission intensity of ThT dye at 480 nm (excitation at 450 nm) increases significantly when it binds to the cross β structure of hydrophobins. (**b**) The morphology detection of the rGf.hyd9954 assembly with AFM. The surface topography of rGf.hyd9954-modified mica was carried out. From the image, we confirmed the rGf.hyd9954, like any class I hydrophobins, is able to self-assemble into amyloid-like fibrils with lengths in the range of 100–150 nm.

HFBs rearrange at interfaces and self-assemble so that they can exhibit a unique and high degree of layout order along with alterations in the secondary structure, such as the dynamic conversion of α-helix and β-sheet [67]. To further characterize the self-assembly process of rGf.hyd9954, a ThT-binding assay was carried out. After the ThT solution was added to the fresh rGf.hyd9954 solution, but without vortexing, the fluorescence intensity was only around 4220 (Figure 4a). The fluorescence intensity at 490 nm exhibited an immediate increase to approximately 13,665 upon vortexing the rGf.hyd9954 and ThT solutions, signifying a marked difference when compared with the non-vortexed group. The observed phenomenon can be attributed to the vortexing treatment of rGf.hyd9954, which creates a gas-liquid interface, facilitates the self-assembly of the HFB, and is accompanied by an increase in β-sheet structure.

### 3.7. The *rGf.hyd9954* Self-Assembly Behavior

The dynamic behavior of HFBs possesses a vital physical significance for the development of macrofungi, i.e., the self-assembly of hydrophobins may initiate the formation of the aerial structure (hyphae and primordia) through breaking the water layer by attenuating the surface tension [4]. Morphological studies revealed a most interesting and striking property of class I HFBs, namely the formation of a highly ordered series of rodlets on the spore surface [68]. Furthermore, rodlet assembly was observed in the self-assembly of membranes at interfaces formed by class I HFBs [69]. We next sought to investigate the morphological features of rGf.hyd9954 via atomic force microscopy (AFM). Intriguingly, the rGf.hyd9954 assembled into polymeric, uniformly, compactly arranged rodlets, which are in accordance with typical class I HFBs (Figure 4b). Our data aligned with those from a published research paper [70], showing that rGf.hyd9954 possesses the ability to self-assemble.

### 3.8. 1,8-ANS Fluorescence Measurements

**Figure 5:**
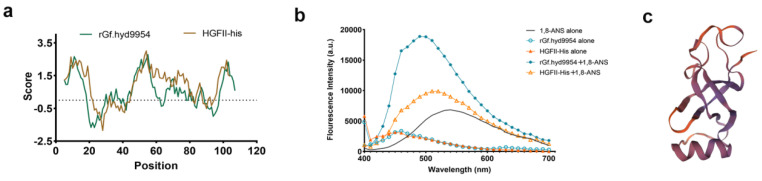
The hydropathy plot analysis, surface hydrophobicity measurements and the putative tertiary structure. (**a**) The hydropathy plot analysis was performed by the Kyte and Doolittle method to analyze the hydrophobicity of proteins. (**b**) The fluorescence spectrum of 1,8-ANS in the presence of *rGf.hyd9954* and the spectrum of HGFII-his. (**c**) The automated protein structure homology modeling of *rGf.hyd9954* on Swiss-model (https://swissmodel.expasy.org/, accessed on 18 September 2023). Homology-based three-dimensional model of the solution NMR structure of a class I hydrophobin from *Serpula lacrymans* (5w0y.1.A).

1,8-ANS is essentially non-fluorescent in water, but it becomes appreciably fluorescent when bound to the non-polar regions of a protein. Therefore, 1,8-ANS was employed here as a highly sensitive probe with which to quantify HFB unfolding. We investigated the hydrophobicity of rGf.hyd9954 via 1,8-ANS. When an HFB was mixed a 1,8-ANS solution, the peak position showed a blue shift, indicating that the HFB aggregated and the hydrophobic region of the protein was exposed. According to the quantification, the fluorescent peak position of rGf.hyd9954 was near 510 nm, which was almost 2.10-fold higher than that of HGFII-his (Figure 5b). It is speculated that the slightly more exposed hydrophobic regions of the rGf.hyd9954 primary structure, compared with HGFII-his, result in a greater ability of the former to interact with 1,8-ANS. Aligned with the expression pattern, WCA, and XPS results, the results of these assays provide strong evidence to validate that rGf.hyd9954 possesses excellent bio-surfactant properties. According to these outstanding traits, it is possible to propose the hypothesis that rGf.hyd9954 might be the main contributor supporting primordia formation and fruiting body development in *G. frondosa*.

### 3.9. The Putative Tertiary Structure of *rGf.hyd9954*

The homology of a putative model of the tertiary structure of rGf.hyd9954 with the structure of a class I HFB, 5w0y.1.A, from *Serpula lacrymans* was compared by NMR (Figure 5c). rGf.hyd9954 shares the α-helix and the β-barrel core of the HFB fold, with the loop regions between the conserved cysteine residues, likely to result in certain degrees of flexibility, and conserved structures of class I HFBs, pivotal to the amphipathic self-assembly into fibrils and involved in the stability of the protein.

### 3.10. The Particle Size and Zeta Potential of *rGf.hyd9954*

**Figure 6:**
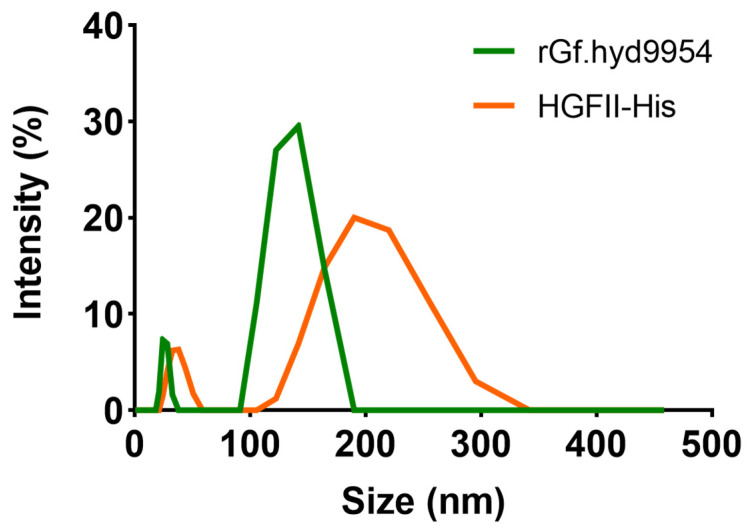
The particle size assay. The particle sizes were carried out by the dynamic light scattering (DLS) method to analyze the particle size of rGf.hyd9954 solutions at 100 μg/mL.

Due to their spectacular self-assembly properties, HFBs can be used as bio-surfactants, co-solvents, and chaotropic agents. Through dynamic light scattering (DLS) measurement, we can determine whether its particle size belongs to the nanoscale, thereby expanding its application fields. The particle size of rGf.hyd9954 was approximately 100–200 nm. With its greater molecular weight, the particle size of HGFII-his was around 100–350 nm (Figure 6). The zeta potential for rGf.hyd9954 was −20.5 ± 1.39 mV; by comparison, the value for HGFII-his was −19.8 ± 0.15 mV. These results specify that the surfaces of HFBs are negative and that rGf.hyd9954 might be more stable in solution than HGFII-his.

### 3.11. The Emulsification Properties of *rGf.hyd9954*

**Figure 7:**
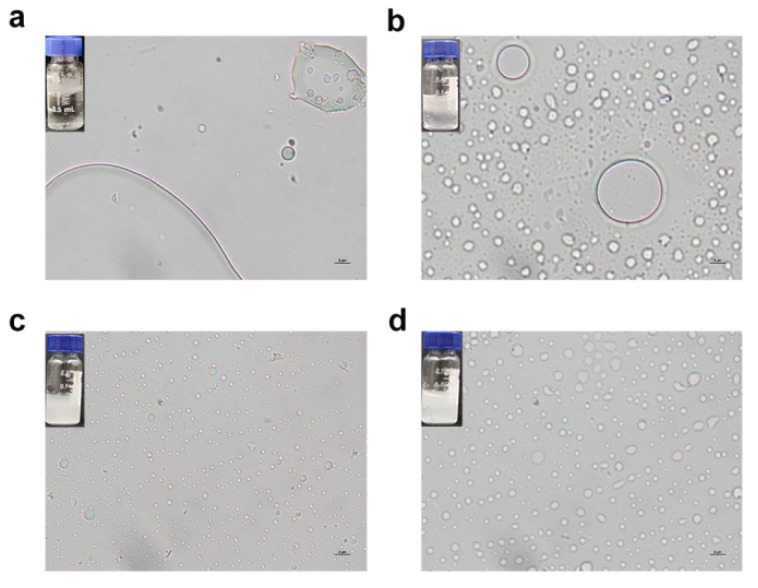
The emulsification properties determination of rGf,hyd9954. (**a**) The ddH2O-soybean oil; (**b**) the BSA solution-soybean oil; (**c**) the rGf.hyd9954-soybean oil; and (**d**) the HGFII-his-soybean oil emulsification systems. The protein concentration of the protein solutions was 100 μg/mL. The insertion figures were the emulsification systems contained in a transparent vial after the homogenization treatment for 72 h, bar 5 μm.

Emulsification is the process of dispersing one liquid into another, the liquids otherwise being either immiscible or water-insoluble. Many studies have focused on the role of emulsification in the oil recovery and cosmetics industries. Given the exceptional amphipathic nature and the surface activity of this novel HFB, an oil emulsification assay was performed to evaluate the emulsification properties of rGf.hyd9954. After allowing soybean oil to distribute in ddH_2_O and stand at room temperature for 72 h, the emulsification system had turned extremely clear, with oil droplets collecting at the top layer of the system (Figure 7). In contrast, the oil emulsification system with rGf.hyd9954 or HGFII-his showed that the oil did not aggregate but was evenly dispersed in the presence of either HFB, maintaining a stable emulsion state. The rGf.hyd9954 oil emulsification system exhibited superb turbidity, and the entire solution appeared opaque and opalescent, showing a better emulsification effect than HGFII-his. When observing the state of droplets under an optical microscope under the same conditions, the number of oil droplets dispersed by HGFII-his was larger and the droplet size was more uniform than for rGf.hyd9954 (the insertion figures in Figure 7). Due to the greater surface hydrophobicity of rGf.hyd9954 than that of HGFII-his, as confirmed by 1,8-ANS fluorescence determination, rGf.hyd9954 was better able to efficiently encapsulate the oil droplets within the self-assemblage when encountering the hydrophobic interface formed by oil droplets, thus forming a relatively stable emulsification system. This observation shows that rGf.hyd9954 would be a good candidate as a naturally occurring emulsifier.

## 4. Conclusions

Overall, we identified 19 typical class I HFB genes from the mycelia of the *G. frondosa* strain CICC^®^50075. The gene sequence and protein primary structure exhibited polymorphisms, although they showed similarities with another *G. frondosa* strain, 9006-11, in phylogenetic relationships. Expression of the HFB genes in *G. frondosa* was developmentally and temporally regulated. Intriguingly, we observed that *Gf.hyd9954* showed marked transcript abundance at the primordia and young fruiting body stages, especially at the former stage, indicating that Gf.hyd9954 was a predominant class I HFB, functioning at specific time points around the formation of the fruiting body. Our better understanding of the diversity and expression pattern of HFBs in *G. frondosa*, resulting from the current study, will undoubtedly be highly beneficial in illuminating how the HFB genes coordinate during the development process of *G. frondosa*, particularly the significance of the predominant hydrophobin Gf.hyd9954 in the fruiting process due to its high surface activity and its ability to self-assemble into nanoscale rodlet bundles. Given that hydrophobins are notable biosurfactants, rGf.hyd9954 was additionally assessed for emulsification properties and shown to possess the potential to be an effective chaotropic agent in pharmaceutical and industrial fields.

However, the current study highlights the need for additional experiments through techniques such as knock-out/knock-down (by CRISPR-Cas9 or RNA interference, respectively) and over-expression of *Gf.hyd9954*, in combination with phenotype characterization, to uncover the physical function of the corresponding HFB during the development of *G. frondosa*. Furthermore, investigating the potential for functional cooperative mechanisms during the development of the mushroom and expanding the range of practical applications for *G. frondosa* hydrophobins will be important from both a theoretical and a practical standpoint.

## Figures and Tables

**Table 1:** Bioinformatic analysis of typical * HFBs in *G. frondosa* CICC^®^50075.

Name	CDS(bp)	DNA (bp)	Number of Introns	Number of Exons	Number of Amino Acids	Molecular Weight (Da)	Class of Hydrophobin
*Gf.hyd2041*	429	2613	3	4	142	14,361.64	I
*Gf.hyd6681*	357	1227	2	3	118	11,782.75	I
*Gf.hyd6682*	342	1692	4	5	113	11,372.04	I
*Gf.hyd7622*	324	2225	2	3	107	10,470.30	I
*Gf.hyd8174*	429	3784	7	7	142	14,106.29	I
*Gf.hyd8531*	324	2445	2	3	107	10,456.27	I
*Gf.hyd8825*	384	1881	2	3	127	12,549.59	I
*rGf.hyd9954*	336	1789	2	3	111	10,917.37	I
*Gf.hyd10182*	303	1601	2	3	100	9723.19	I
*Gf.hyd11240*	1179	2562	2	3	392	36,493.27	I
*Gf.hyd11347*	1131	2510	3	4	376	35,047.55	I
*Gf.hyd12081*	417	1040	1	1	138	13,564.40	I
*Gf.hyd12082*	699	1976	3	4	232	23,671.27	I
*Gf.hyd12802*	429	2157	2	3	142	13,949.09	I
*Gf.hyd13942*	339	2051	2	3	112	11,241.08	I
*Gf.hyd14947*	312	1594	2	3	103	10,078.58	I
*Gf.hyd15024*	420	1817	4	4	139	13,846.22	I
*Gf.hyd20923*	321	596	3	3	106	10,708.47	I
*Gf.hyd21629*	312	548	2	3	103	9782.11	I

Typical * represents the hydrophobins contain eight conserved cysteine residues that are displayed in a distinct pattern (X_2–38_-C-X_5–9_-C-C-X_11–44_-C-X_8–23_-C-X_5–9_-C-C-X_6–18_-C-X_2–14_), share a characteristic hydropathy profile, and are abundant in hydrophobic residues.

**Table 2:** Physico-chemical characterization of typical HFBs in *G. frondosa* CICC^®^50075.

Name	Sub-Location *	Theoretical PI	GRAVY **	Signal Peptide	Number of Negative Charged Residues	Number of Positive Charged Residues	GO *** Terms
Gf.hyd2041	plas	8.50	0.270	Yes	7	10	9
Gf.hyd6681	extr	3.57	0.858	Yes	11	1	9
Gf.hyd6682	extr	6.05	0.441	Yes	5	5	null ****
Gf.hyd7622	extr	4.04	0.951	Yes	5	2	9
Gf.hyd8174	extr	7.52	0.336	Yes	8	9	9
Gf.hyd8531	extr	4.04	0.950	Yes	5	2	9
Gf.hyd8825	extr	4.00	0.876	Yes	4	1	9
rGf.hyd9954	extr	3.62	0.495	Yes	7	1	9
Gf.hyd10182	extr	5.30	0.723	Yes	2	1	9
Gf.hyd11240	extr	4.87	0.368	Yes	14	8	9
Gf.hyd11347	extr	5.31	0.343	Yes	13	8	9
Gf.hyd12081	extr	4.01	0.675	Yes	9	3	null
Gf.hyd12082	extr	4.31	0.524	No	17	12	9
Gf.hyd12802	extr	4.46	0.777	Yes	8	5	9
Gf.hyd13942	extr	3.57	0.789	Yes	7	1	9
Gf.hyd14947	extr	5.30	0.682	Yes	2	1	9
Gf.hyd15024	extr	3.98	0.878	Yes	8	3	9
Gf.hyd20923	extr	4.99	0.644	Yes	6	5	null
Gf.hyd21629	extr	3.19	0.818	No	8	0	9

Plas * represents plasma; Extr represents extracellular. GRAVY ** indicates grand average of hydropathicity. GO *** indicates gene ontology term; GO_BP: Biological processes gene ontology term; GO_CC: Cellular component gene ontology term and GO_MF: Molecular function gene ontology term. Null **** represents no data from the GO data source.

**Table 3:** The water contact angle (WCA) measurements of coating Teflon film and mica slices.

Hydrophobins	Processing	WCA
	Blank Teflon film	86.32 ± 3.26°
	Blank mica slice	8.86 ± 0.63°
rGf.hyd9954	a	59.48 ± 2.47°
	a + c	62.17 ± 4.15°
	b	20.73 ± 0.98°
	b + c	17.43 ± 2.19°
HGFII-his	a	58.40 ± 2.71°
	a + c	71.79 ± 2.56°
	b	17.38 ± 0.57°
	b + c	7.22 ± 0.45°

The coating surface was rinsed with a hot 2% SDS solution, respectively, which followed the WCA measurements to evaluate the wettability alteration capability and resilience profile of the coating film of rGf.hyd9954. a, coating teflon film; b, coating mica slice, c, rinsed with a hot 2% SDS solution.

## Data Availability

Data are contained within the article and Supplementary Materials.

## Supplementary Materials

The following supporting information can be downloaded at: https://www.mdpi.com/article/10.3390/jof10010025/s1, Figure S1. The cloning of *rGf.hyd9954* in *P. pastoris* and the confirmation of the recombinant strains. Figure S2. The validation of the recombinant rGf.hyd9954. Figure S3. The COG (Clusters of Orthologous Groups) analysis of the *G. frondosa* CICC^®^50075. Figure S4. The water contact angle (WCA) measurements of coating Teflon film and mica slices. Table S1. The amino acid sequences of the identified hydrophobins of *G. frondosa* CICC^®^50075 in this study. Table S2. The CDS (coding sequence) of the identified hydrophobins and the *gapdh* gene of *G. frondosa* CICC^®^50075 in this study. Table S3. The primers for qRT-PCR of typical hydrophobin genes used in this study. References [71,72,73,74,75,76,77] are cited in the Supplementary Materials.
