# Supplementary material for: Identification and Characterization of a Predominant Hydrophobin in the Edible Mushroom Grifola frondosa
Source: J Fungi (Basel). 2023 Dec 29;10(1):25. doi: 10.3390/jof10010025 (PMC10820438; doi:10.3390/jof10010025)
Supplement: Supplementary file 1 [file jof-10-00025-s001.zip › jof-2765512-supplementary.pdf]

Supplementary Materials

(Additional experimental details, methods and data)

Identification and Characterization of a Predominant Hydrophobin in the Edible Mushroom *Grifola frondosa*

Supporting information for Materials and Methods

1. Illumina sequencing and de novo assembly

1.1 Extraction of genome DNA

Genomic DNA was extracted with the SDS method[1]. The harvested DNA was detected by the agarose gel electrophoresis and quantified by Qubit 2.0 Fluorometer (Thermo Scientific).

1.2 Library construction

A total amount of 1 μg DNA per sample was used as input material for the DNA sample preparations. Sequencing libraries were generated using NEBNext® Ultra™ DNA Library Prep Kit for Illumina (NEB, USA) following manufacturer's recommendations and index codes were added to attribute sequences to each sample. Briefly, the DNA sample was fragmented by sonication to a size of 350 bp, then DNA fragments were end-polished, A-tailed, and ligated with the full-length adaptor for Illumina sequencing with further PCR amplification. At last, PCR products were purified (AMPure XP system) and libraries were analysed for size distribution by Agilent2100 Bioanalyzer and quantified using real-time PCR.

1.3 Sequencing

The whole genome of *G. frondosa* was sequenced using Illumina HiSeq PE150 platform at the Beijing Allwegene Technology Co., Ltd.

1.3 Genome assembly

All good quality paired reads were assembled using the SPAdes(v3.13.0)[2]

software into a number of scaffolds. Finally, scaffolds with larger than 200 bp were selected for subsequent analysis.

1.4 Genome component prediction

Genome component prediction included the prediction of the coding gene, repetitive sequences, non-coding RNA, protein signal peptide, secretory protein and pseudogene. The available steps were proceeded as follows: 1) We used the Prokka (1.13.7)[3] software to retrieve the related coding gene. 2) The interspersed repetitive sequences were predicted using the RepeatMasker (v4.0.9). 3) Transfer RNA (tRNA) genes were predicted by the tRNAscan-SE[4]. Ribosome RNA (rRNA) genes were analyzed by the rRNAmmer (v1.2)[5]. 4) Genewise[6] was used to identify the pseudogene.

1.5 Gene function

We used 7 databases to predict gene functions. They were respective GO (Gene Ontology, http://geneontology.org/), KEGG(Kyoto Encyclopedia of Genes and Genomes, https://www.kegg.jp/), COG(Clusters of Orthologous Groups, https://www.ncbi.nlm.nih.gov/research/cog-project/), NR(Non-Redundant Protein Database databases), Swiss-Prot(https://www.uniprot.org/), CARD(the Comprehensive Antibiotic Research Database, https://creativecommons.org/licenses/by/4.0/), Pfam(http://pfam.xfam.org/), and InterproScan(http://www.ebi.ac.uk/interpro/). Carbohydrate-Active enzymes were predicted by the Carbohydrate-Active enZYmes Database

(http://www.cazy.org/).

2. **Heterologous expression of hydrophobin in *Pichia Pastoris***

2.1 Cloning

Herein, we report the heterologous soluble expression of the recombinant class I hydrophobin Gf.hyd9954 originating from *G. frondosa*, and its efficient purification from recombinant *Pichia Pastoris* GS115. Efficient expression of the recombinant hydrophobin Gf.hyd9954 was achieved by a tagging strategy using a 6*His tag that was fused to the C-terminus of Gf.hyd9954 lacking the innate signal sequence. Subsequently the recombinant hydrophobin Gf.hyd9954 in a soluble form was efficiently purified by an immobilized metal affinity chromatography (IMAC) technique using High-Affinity Ni-Charged Resin FF (GenScript®, Nanjing) columns.

The DNA sequence corresponding to the amino acid sequences coding for Gf.Hyd9954 (Table S1) without native signal peptide were codon optimized, synthesized and DNA sequencing were performed by Genewiz®(Suchou, China). The DNA cassette of *rGf.hyd9954* was inserted in the pPIC9K vector (9276 bp) between the *Eco*RV and *Not*I restriction sites obtaining the recombinant plasmid pPIC9K-*rGf.hyd9954.* The plasmids construction schematic diagrams is shown in Fig. S1a. Subsequently, the recombinant plasmid was propagated in *E. coli* DH5α. The recombinant plasmid extraction was performed according to the manufacturer's instruction (TIANprep Mini

Plasmid Kit, TIANGEN, China) and linearised with *Sac*I, then examined by agarose gel electrophoresis (Fig. S1b). Additionally, the purified recombinant plasmids were electro-transformed into *P. pastoris* GS115 competent cells using a gene pulser apparatus (MicroPulser™, Bio-Rad Laboratories, USA) and a 2 mm electroporation cuvette, a sample volume of 100 μl, a charging voltage of 1.5 kV and a pulse length of about 5 ms. The *E. coli* recombinant strain were cultivated in Luria broth (LB) (yeast extract 5 g/L, tryptone 10 g/L, NaCl 10 g/L) medium at 37°C for 16 h, while the transformed colonies were spread and selected on MD agar plates and incubated at 30 °C for 36 - 48 h, and the recombinant *P. pastoris* strain was cultured in buffered minimal medium (BMM) (for shake flask fermentation) at 28°C for 96 h for obtaining the interest protein[7]. The heterologous expression hydrophobin was named as rGf.hyd9954 with 9.94 kDa molecular weight.

2.2 Validation of transformants

A PCR screening approach of putative transformants was performed to validate the homologous integration of the *rGf.hyd9954-his* gene cassette (279 bp) into downstream of the *AOX1* in the *P. pichia* GS115 genome. The primers used were specific to the *aox1* gene (*AOXI* F: 5'-GCAAATGGCATTCTGACATCC-3', *AOX1* R: 5'-GACTGGTTCCAATTGACAAGC-3'), sequences flanking the *AOX1* cassette. The products were analyzed by horizontal gel electrophoresis on 1% (w/v) agarose gel (Fig. S1c).

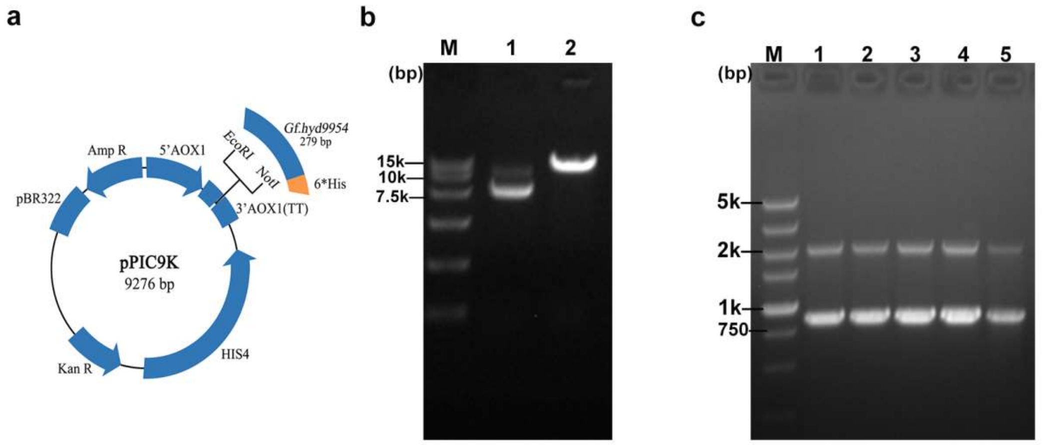

Figure S1. The cloning of *Gf.hyd9954* in *P. pastoris* and the confirmation of the recombinant strains. (a) The schematic diagram of plasmid constructs. The pPIC9k-*Gf.hyd9954* was synthesised by GENEWIZ® cooperation(Suchou, China). (b) The validation of *Sac*I digested product of plasmid pPIC9k-*Gf.hyd9954.* M: DNA standard marker. Lane 1: natural plasmid pPIC9k-*Gf.hyd9954*; lane 2: *Sac*I digested plasmid pPIC9k-*Gf.hyd9954.* (c) The PCR conformation of *AOX1-Gf.hyd9954* cassette. M: DNA standard marker. Lane 1-5: transformants 1-5. The 2.2 kb-band were the AOX1 cassette exits in the *P. pastoris* genome, the 792 bp-band were the *Gf.hyd9954* gene linked with the AOX1 flanking sequence. The two bands indicated that the transformants were the methanol utilizing GS115 recombinant strains with inserting gene.

2.3 Protein production and purification

The high yield *P. pastoris* recombinant strain was cultivated in BMM media in shake flasks, adding 0.6% (v/v%) methanol at an interval of 24 h to obtain rGf.Hyd9954. The fermentation was stopped after 96 h, after which the

fermentation broth was harvested by centrifugation for 10 min at 6000 g, 4 °C (Eppendorf Centrifuge 5810R), to harvest the supernatant. The purification process was according to the procedure described in a previous study[7]. Finally, desalting and concentration of the rGf.Hyd9954 were performed by centrifugation (Eppendorf Centrifuge 5810R, 3500 g, 60 min for 5 times) with ultrafiltration tubes (AMICON ULTRA 3 K NMWL 96PK, Merck). The purified protein was lyophilized and stored in a dry environment at room temperature, followed by silver staining and western-blotting identification. Protein concentration was quantified using a BCA Protein Assay Kit (Cowin Bio, Suchou, China). The target protein were verified by 12% sodium dodecyl sulfatepolyacrylamide gel electrophoresis (SDS-PAGE) using western-blotting (Fig. S2a) with anti-His-tag HRP mAb (GenScript, Nanjing). The time courses of fermentation (Fig. S2b) and the purification were validated via silver staining (Fig. S2c). The 96 h of fermentation was the optimal fermentation time.

The molecular weight of the recombinant rGf.hyd9954 containing the C-terminal 6*His was 9.94 kDa. According to the Western-blotting and silver staining analysis of the fermentation broth and specimen of the purified product, the bands of ~10 kDa indicated the rGf.hyd9954 was successfully expressed in *P. pichia*, and the optimal fermentation time was 96 h. In addition, the rGf.hyd9954 was successfully purified via the Ni-affinity chromatography.

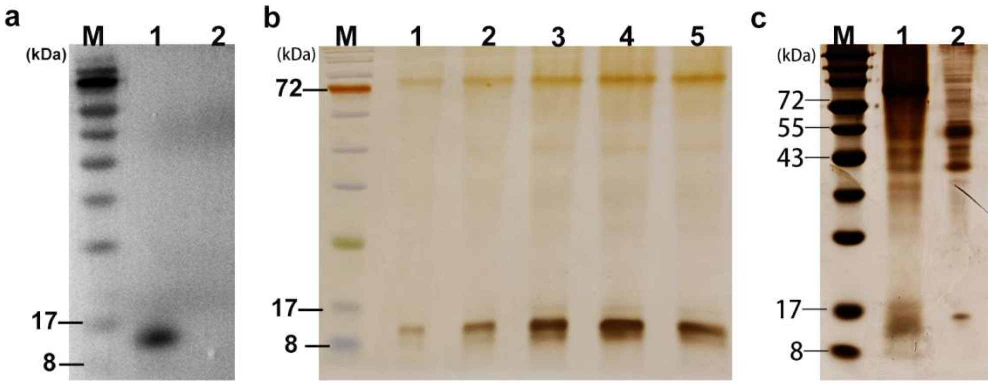

Figure S2. The validation of the recombinant rGf.hyd9954. (a) The flask fermentation specimen were checked via western-blotting, lane 1 was the fermentation sample and lane 2 was the negative control. (b) The silver-staining analysis of the time courses of the flask fermentation. M: Molecular weight (MW) standard marker. Lane1-5 were the 48 h, 72 h, 84 h, 96 h and 108 h, respectively. (c) The Ni-affinity chromatography and ultrafiltration centrifugation was carried out to purify the recombinant rGf.hyd9954. Lane 1 was the fermentation supernatant and lane 2 was the purified product.

3. The basic information of the *G. frondosa* genome

By the shotgun genome sequencing of *G. frondosa* CICC®50075, 21318 scaffolds were identified and the GC content was 50.96%. The whole genome of *G. frondosa* CICC®50075 contains 22429 genes, which was 49% more than that of another *G. frondosa* linage, 9006-11 (NCBI: Whole Genome Shotgun (WGS): INSDC: LUGG00000000.1), however, with similar GC content around 50%.

Due to the the *G. frondosa* is the Basidiomycetes, which possess the varied

life cycles, they are able to produce an incredibly diverse classifications of metabolic compounds[8]. Thus, we performed COG（Clusters of Orthologous Groups）analysis to have a overall understanding of the metabolic profiles of the *G. frondosa.*

Overall, the COG data shows 513 genes were matched for the *General function prediction only* cluster, then the second large cluster is the *Carbohydrate transport and metabolism*, containing 366 genes, followed by the *Amino acid transport and metabolism* cluster, which contains 331 matched genes. Intriguingly, 284 genes involved in the *Secondary metabolites biosynthesis, transport and catabolism* cluster, which further indicating the powerful biosynthetic properties of the *G. frondosa.* Moreover, 91 genes were involved in the *transcription* process according to the COG data (Fig. S3).

As for the genes involves in transcription regulation or transcription regulation, we found that there were 55 genes in the GO analysis matched these functions, and 27 genes were related to the *transcription factor TFIID/TFIIA/TFIIE/F complex.* Besides, 4 genes were clustered to the *mitochondrial genome maintenance* function in the GO enrichment results. We believe these unigenes were responsible and possess great importance for the growth and development of *G. frondosa*.

The genome sequencing data of this work will be submitted online in the near future.

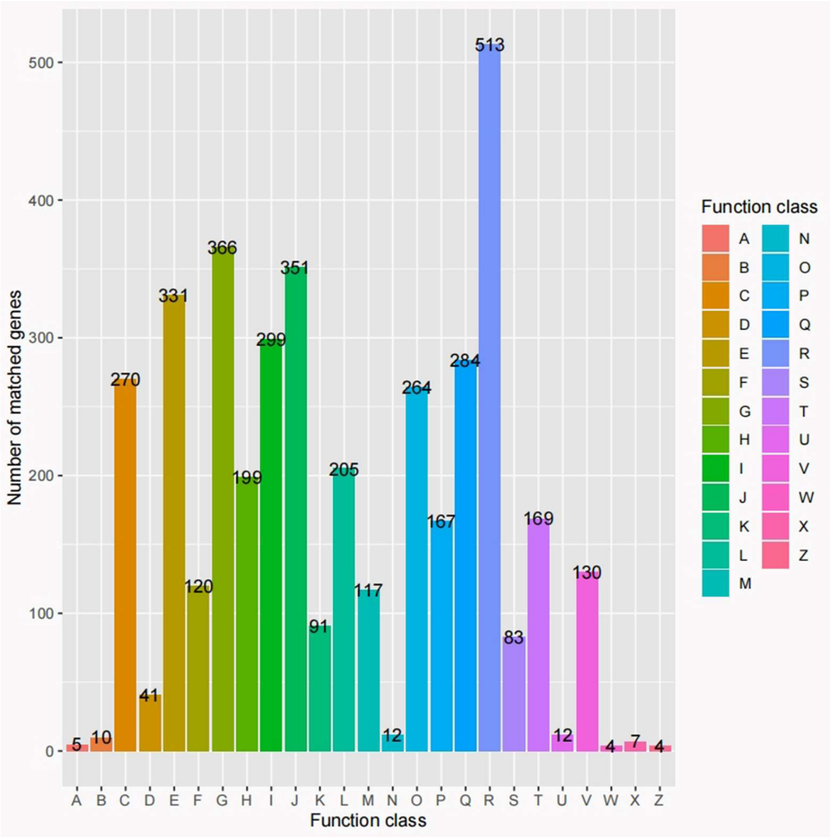

Figure S3. The COG（Clusters of Orthologous Groups）analysis of the *G. frondosa* CICC®50075. A:RNA processing and modification; B:Chromatin structure and dynamics; C:Energy production and conversion; D:Cell cycle control, cell division, chromosome partitioning; E:Amino acid transport and metabolism; F:Nucleotide transport and metabolism; G:Carbohydrate transport and metabolism; H:Coenzyme transport and metabolism; I:Lipid transport and metabolism; J:Translation, ribosomal structure and biogenesis; K:Transcription; L:Replication, recombination and repair; M:Cell wall/membrane/envelope biogenesis; N:Cell motility; O:Posttranslational modification, protein turnover, chaperones; P:Inorganic ion transport and metabolism; Q:Secondary metabolites biosynthesis, transport and catabolism; R:General function prediction only
S:Function unknown; T:Signal transduction mechanisms; U:Intracellular trafficking, secretion, and vesicular transport; V:Defense mechanisms; W:Extracellular structures; X:Mobilome: prophages, transposons; Z:Cytoskeleton.

4. Water contact angle (WCA) determination

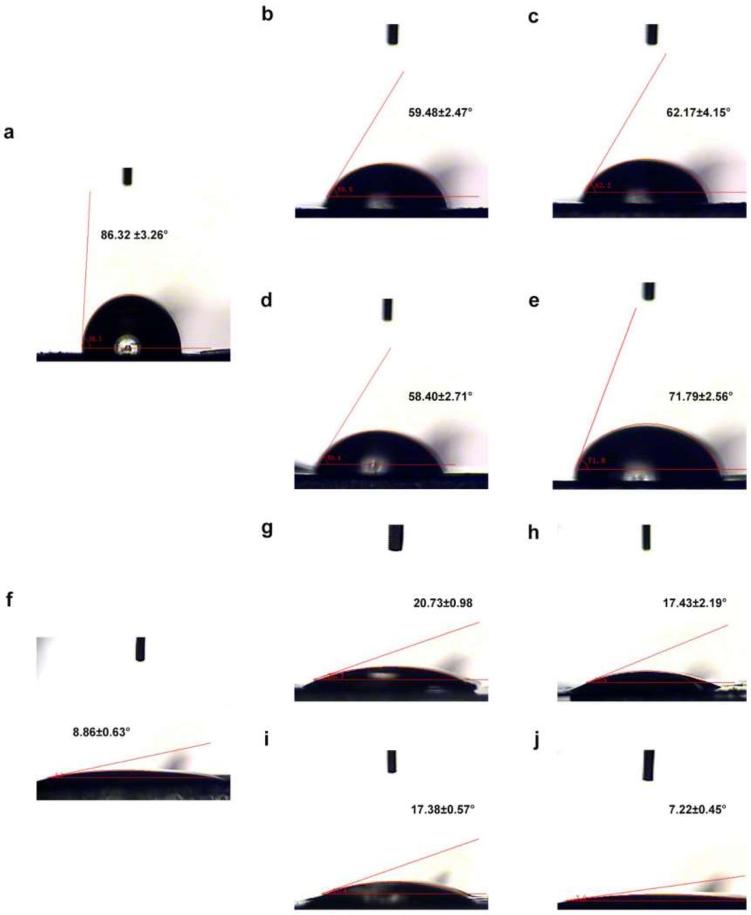

Figure S4. The water contact angel (WCA) measurements of coating Teflon film and mica slices. The coating film was rinsed with hot 2% SDS solution respectively followed the WCA measurements to evaluate the wettability alteration capability and resilience profile of the coating film of rGf.hyd9954. (a). the WCA on the blank Teflon film; (b), the WCA on the rGf.hyd9954 coating Teflon film; (c), the WCA on the rGf.hyd9954 coating Teflon film after rinsed by hot 2% SDS solution; (d), the WCA on the HGFII-his coating Teflon film; (e), the WCA on the HGFII-his coating Teflon film after rinsed by hot 2% SDS

solution; (f), the WCA on the blank mica slice; (g), the WCA on the rGf.hyd9954 coating mica slice; (h), the WCA on the rGf.hyd9954 coating mica slice after rinsed by hot 2% SDS solution; (i), the WCA on the HGFII-his coating mica slice; (j), the WCA on the HGFII-his coating mica slice after rinsed by hot 2% SDS solution.

Table S1. The amino acid sequences of the identified hydrophobins of *G. frondosa* CICC®50075 in this study.

Name	Cys spacing pattern	Amino acid sequence
Gf.hyd 2041	C-X6-CC-X31-C-X13-C-X5-CC-X12-C-X5	MKLTFALTALLAAVISVEATSTNGERLARGLPPMPPRKRYLPSRMEHARRTSPSGSPSSGGSCNTGPIQCCNQTTTADDPVAALLLGLLGIVLGADVPVGLQCSPLSVVGVGSGSACSARPVCCENNSNGSLISIGCIPITL
Gf.hyd 6681	C-X8-CC-X32-C-X12-C-X8-CC-X10-C-X17-C	MLAKLSLLVTTSLALLAAAQSTCDVVSDGLLCCDDLEPGDAPILGPIFQLLGIPLPGIDTIVGLNCSSTNITGILNGACAVGTGHLSCCDINLDIVAIGCQNPTLPSAEALSVLGCDC
Gf.hyd 6682	C-X8-CC-X13-C-X12-C-X6-CC-X11-C-X34	MLAKLALLTTTSFALLATAQNCSVLSTNLLCCEQTQSGGTITGLNCSSTNLAAILLGGCLTGNLACCDNNGSGIGGFECEAPALLPSAVLSVLGSSNQLIFGSARRQSARSEK
Gf.hyd 7622	C-X6-CC-X32-C-X12-C-X5-CC-X12-C-X5	MFSRLATFGFLALPLLATATPLMPRDSCDTGPIQCCATTETAGSASGAAILGLLGIVIQDLSVLLGVGCSPISVIGVGGGTCDASPVCCTNNNVGGLISIGCVPVTL
Gf.hyd 8174	C-X6-CC-X31-C-X13-C-X5-CC-X12-C-X6	MKLTVALAALALTAASVESASLGTNAERFARGLPPLPPRRRYDPTAVSPAKRASPSGSPGQCNTGPIQCCDTVTTANNPVAALILDLLGIVLGPGVAVGLTCSPISVIGVGSGSQCSAHPVCCENNSEGGLISIGCIPKDAL
Gf.hyd 8531	C-X6-CC-X32-C-X12-C-X5-CC-X12-C-X5	MFSRLATFGFLALPLLATATPLMPRDSCDTGPIQCCASTETAGSASGAAILGLLGIVIQDLSVLLGVGCSPISVIGVGGGTCDASPVCCTNNNVGGLISIGCVPVTL
Gf.hyd 8825	C-X6-CC-X32-C-X13-C-X5-CC-X12-C-X5	MFSRTVAFFYFLLSLSVLAVAMPGGAPPTQTVTVTAPASTVTSAGQCNVNDIQCCNSVQSASSGLVSLLEGLLGIVLGPIEGLIGLGCSPISVIGVGSGSECNASPVCCTNNNVGGLISIGCVPITL
Gf.hyd 9954	C-X6-CC-X32-C-X13-C-X5-C	MSSKLTLVLSTLAVLATANPTPDEPASSCNTAPIQCCESVQPASSGVAAALLASVGVVVQDPTTPIGITCSPISDFGVGSGSTCDASPV

	C-X12-C-X7	CCENNSYGSLVSIGCIPVNLQS
Gf.hyd 10182	C-X6-CC-X29-C-X13-C-X5-C C-X12-C-X5	MFARIAAVSFLALAAATSANSQCNTGPIQCCQSANSAAGTALLSMLGVVLNDPTVLIGGQCSPISAVGVGSGSECNAHPVCCTNNNVGGVLSVGCVPVQL
Gf.hyd 11240	C-X6-CC-X32-C-X12-C-X5-C C-X12-C-X5	MQFKLSLISAVTLAVLASASPLAIHKKCDSSSSPADSTWGVPTPTASLSATLIATPPTGSLSAVPTSGVGIGVGVALPGFESGSSAPGGSLPGLSPPVGVLPVGALPGIAAPGVPTGVALGVTLPILGSGSSSAPGSSLPGVSLPGGSLPGSLPGVPLPGSSTPGSSAPGGIDVGVALPGASAPVGSIPGSPAPGVPAGVAVGVNLPGLESGGSSAPGSSVPGIPAPGIPAAVPAGVSLTVALPFGGASTPTPSSPTGQSPTSHASISVSIPTAVPTYSIPAWSDSVPVPSPTAGSSSPHHSGSSDDGSTSQCNTGSIQCCNKFGDASSDDMRSFLLGIGAPLKGLSGLVGAQCSPIDPAGLGNGECKQAPVCCKNDAQGGLVSIGCVPITL
Gf.hyd 11347	C-X6-CC-X32-C-X12-C-X5-C C-X12-C-X5	MQFKLSLISAVTLAVLASASPLAIHKKCDSSSSPAHSTWGAPTPTASLSATLTATLPTGSLSAVPTSGVGIGVSVGGLPGLESGSSAPGGSLPGLSVPVGLPGTPLAGVALGVTLPILGSGSSSAPGSSLPGVSLPGSLPGVSLPGSSTPGSSAPGGIDVGVALPGASAPVGSIPGSPAPGGPAGVAVGVNLPGLESAGSSAPGSSVPGIPAPGIPAAVPAGVSLTVALPFGGASTPTPSSPTGQSPTSHASISVSIPTAVPTYSIPAWSDSVPVPSPTAGSPHHSGSSDDGSTSQCNTGSIQCCNKFGDASSDDMRSFLLGIGAPLKGLSGLVGAQCSPIDPAGLGNGECKQAPVCCKNDAQGGLVSIGCVPITL
Gf.hyd 12081	C-X6-CC-X32-C-X13-C-X5-C C-X12-C-X5	MFSRFSAIFVSTVALSVLATATPNYEAKRWSTSSTAEATTTVTVTATAPASTESAGSCNVGDLQCCNSVESASSPAATTLLGLLGIVVDGLDVLLGLGCSPISVIGVGSGSACDASPVCCENNNVGGLISIGCAPVIL
Gf.hyd 12082	C-X6-CC-X32-C-X12-C-X5-C C-X12-C-X5	MSAYGDVVNFESAPLVAVLRTRILDARTSARIAPADITRTEMQISLLPEGAGHAPSLCRYINPQFELSNSPDRPAALLHTLVPLTILTANMFSRFSAVFVATVALSVLATATPAKRWSTTAATTAKVTTTAKVTTTVTVTATAPASTESAGSCNVGDIQCCDSVESADSPAAVGLLGLLGIVVDGLDVLLGLNCSPLSIIGIGGGSCDANPVCCENNNVGGLISIGCIPIIL
Gf.hyd 12802	C-X6-CC-X32-C-X12-C-X5-C C-X12-C-X5	MFSRFSAVFVATVALSVLATATPAKRWSTTAATTAKVTTTAKVTTTVTVTATAPASTESAGSCNVGSLQCCDSVESADSPAAVGLLGLLGIVVDGLDVLLGLNCSPLSIIGIGGGSCDANPVCCENNNVGGLISIGCIPIIL
Gf.hyd 13942	C-X8-CC-X32-C-X12-C-X6-C C-X12-C-X7	MFGNRALFATTSLALLAAAIPQSTPTQCDFLTSPLLCCQQVLSGDLPILGPILQSLGIPLLGAGTEVGLGCSSTNSIEVLLGACATGDVVCCDNNDFGGIIALGCQNVTGLL
Gf.hyd 14947	C-X6-CC-X32-C-X13-C-X5-C C-X12-C-X5	MFARIAAVSFLALAAATSANSQCNTGPIQCCQSVQQANSAAGTALLSMLGVVLNDPTVLIGGQCSPISAVGVGSGSECNAHPVCCTNNNVGGVISVGCVPVQL
Gf.hyd 15024	C-X6-CC-X32-C-X13-C-X5-C	MFKLSRALVLFCFALSLALLAVARPGASPPDCTTTSFPPPTTTVTVTAPAPTPTGTDQCNTGNIQCCDSSSSPVGSLILGLLGIVVDLV

	C-X12-C-X5	DGVDVLLGLGCNAIVVVGVGESPQCAATPVCCENNSVGGLISIGCIVIIL
Gf.hyd 20923	C-X8-CC-X13-C-X12-C-X6-CC-X11-C-X15	MMRGRSDVIQSIMFAKRALLTTTSFVLLATAQNCSILSTNLLCCDETQSGGTITGLNCSSTKLAAILLGGCLTGNLACCDSSGSGIGGFECEAPALLPSAVLSVLG
Gf.hyd 21629	C-X6-CC-X32-C-X13-C-X5-CC-X12-C-X5	AEATTTVTVTATAPASTESAGSCNVGDLQCCNSVESASSPAATTLLGLLGIVVDGLDVLLGLGCSPISVIGVGSGSACDASPVCCENNNVGGLISIGCVPVIL
Gf.hyd 15802	C-X43-C-X32-C-X13-C-X5-CC-X12-C	MFKLSRVLALFCFALSLALLAVARPGASPPDCTTTTSFPPPTTTVTVTAPAPTPTGTDQHVRPAHPGSPLLTSYFCAQSSSPVGSLILGLLGIVVDLVDGVDVLLGLGCNAIVVVGAGESPQCAATPVCCENNSVGGLISIGCIVIIL (7*Cys)
Gf.hyd 19717	C-X6-CC-X32-C-X13-C-X5-CC-X5	MFSRFSALFVSTVALSVLAVATPNPVKRGGEPTTTTVKATTTITVTATAPAASESAGSCNVSPIQCCESTEIASSAAGTTLLGLLGIVLTDLNVLLGLNCSPLSIIGVGSGSACDASPVCCENNNV (7*Cys)
Gf.hyd 4500	C-X8-CC-X32-C-X12-C-X10-C-X12	MTSLALLAAANPAPVPQSSAEGCTLLVDNLLCCQEFEPGDAPILGPIFELLGLLLPGVNTLVGLQCTSTNAVEVALGSCLDGNTSANSVCAQEESLPLDAIL (6*Cys)

The eight conserved cysteine residues are highlighted in yellow. The hydrophobin in blue were the truncated hydrophobins that contain 7 or 6 Cys residues.

Table S2. The CDS (coding sequence) of the identified hydrophobin genes and the *gapdh* gene of *G. frondosa* CICC®50075 in this study.

Name	CDS sequence
Gf.hyd2041	atgaagctcacattcgctctgactgccctccttgctgccgtcatctctgttgaggcaacttcgaccaacggc<br>gaacgccttgcccgcggcttgccgcctatgccacctcgcaagcggtatctcccttcgcgcatggaacatg<br>caaggaggacgtctccttctggcagcccttcgtccggaggctcttgcaacactggtcccatccagtgctg<br>caatcagacgaccaccgctgatgatcctgtcgccgccttgctcctcggattgcttggcattgtcctgggtg<br>ctgatgtccccgttggcctccagtgctctcctctcagcgtagttggcgtcggcagtggcagcgcttgctcc<br>gccagacctgtctgctgcgagaacaacagcaacggctcgctcatttccattggttgcatccccatcacact<br>ctga
Gf.hyd6681	atgctcgcaaaactctcgttgctcgtaacgacctcccttgcacttcttgcggccgcccaatcgacctgcgat<br>gtcgtctccgacggcctcctctgctgcgacgatctcgagccgggggatgcacccattctgggccccatct<br>tccagttgctcggaataccgctccctggaatcgacaccattgttgggcttaactgttcgtcgaccaacatc<br>actggaatcttgaacggcgcctgtgccgtcggaactgggcacctgtcgtgttgcgacataaatctcgata<br>ttgtggcgattggatgccaaaacccgaccctaccgtccgctgaggcgttgagcgtattgggatgcgact<br>gctga
Gf.hyd6682	atgcttgccaaacttgcactcctcactacaacctccttcgcgctccttgcgaccgcccagaattgcagcgtt<br>ctgtcaaccaaccttctctgttgcgagcagacccagtccggcggaaccattactgggctcaactgctcgtc<br>gaccaatctggcggctatcctgctgggggggttgtctcactggaaacctggcctgctgcgacaataatggt<br>tcggggattggcgggtttgaatgcgaggctccggccttattaccatctgcagtattgagcgttttgggttc

	ctccaatcagttgatcttcgggtctgctcgccgacaaagcgctcgaagtgagaaataa
Gf.hyd7622	atgttctctcgtcttgccaccttcggattccttgccctcccgcttcttgcgaccgccactcccctcatgccccgt<br>gatagctgcgacaccggccccatccagtgctgtgcgaccactgagacggctggatccgcttcgggggc<br>tgccatcctgggccttctcggtatcgttattcaggatttgagcgtcctcctcggcgtgggctgctccccgat<br>ctccgtcattggcgttggcggcgggacttgcgatgccagcccggtctgctgcacgaacaacaacgtgg<br>gcggcctgatctccatcggctgcgtccccgtcactctctaa
Gf.hyd8174	atgaagctcaccgtcgccctcgccgccctcgctctgaccgccgcttccgtcgaaagcgcatccctcggta<br>ccaacgccgagcgctttgcacgcgggcttcccctctgcccccgcgcagacggtacgatcctaccgctgt<br>ctcccctgcgaagagggcgtcgccatcgggctccccgggccagtgcaacacaggacccatccagtgct<br>gtgacactgtgacgaccgcaaacaaccccgtcgccgcgctcatcctcgacctcttgggcattgtccttgg<br>acccggtgtcgccgtcggcctcacatgctcgcccatctctgttattggggtgggcagcgggagccagtg<br>ctctgcgcatccggtgtgttgcgagaataacagcgagggcgggttgatctccattggatgcattcccaaa<br>gacgccctgtaa
Gf.hyd8531	atgttctctcgtcttgccaccttcggattccttgccctcccgcttcttgcgaccgccactcccctcatgccccgt<br>gatagctgcgacaccggccccatccagtgctgtgcgagcactgagacggctggatccgcttcgggggc<br>tgccatcctgggccttctcggtatcgttatccaggatttgagcgtcctgctcggcgtgggctgctccccgat<br>ctctgtcattggcgttggcggcggcacttgcgatgccagcccggtctgctgcacgaacaacaacgtggg<br>cggcctgatctccatcggctgcgtccccgtcactctctga
Gf.hyd8825	atgttctcccgcaccgtcgccttcttctacttcctgctctcgctctccgtccttgcggtcgccatgcccggcgg<br>cgccccgcccacgcagaccgtcacggtgaccgcgcccgcgtcgaccgtcacctccgccggccagtgca<br>acgtcaacgatatccagtgctgcaacagcgtccagagcgcctccagcgggctcgtgagccttctcgagg<br>gcctgctgggcatcgtgctgggccccatcgagggcctcataggcctggggtgctcgccgatctccgtca<br>tcggcgtcggctccggcagcgagtgcaacgcctccccggtctgctgcacgaacaacaacgtcggtggg<br>ctcatttcgatcggttgcgtccccatcactctctaa
Gf.hyd9954	atgtcctccaagctcacactcgtcctttccactcttgccgtccttgccaccgctaatcccacccccgacgagc<br>cggcgagctcctgcaatactgcgcccatccagtgctgtgaatccgtgcagcccgctagcagcggggtg<br>gcagctgccctgctcgcctcagttggcgtcgttgtccaggatcccaccacacctatcggtatcacttgctct<br>cccatctctgacttcggcgtcggaagtggaagcacctgcgatgcttctcctgtgtgctgcgaaaataattc<br>ttatggtagcttggtgtcgatcggctgcattccggtcaaccttcaatcataa
Gf.hyd10182	atgttcgcccgcatcgctgctgtttccttcctcgcccttgcggctgccacctctgctaactcgcagtgcaaca<br>ccggtcccatccagtgctgccagagtgcgaactccgctgctggcaccgctcttctctccatgttgggcgtt<br>gtgctgaatgatccgactgtgttgattggtggccaatgctcgcccatctctgccgttggcgttggtagcgg<br>ctcggagtgcaatgcgcaccctgtgtgctgcactaacaacaatgttggtggagtgctttctgttggctgtg<br>ttcccgtccagctctaa
Gf.hyd11240	atgcagttcaagctctccctcatctccgccgtcaccctcgccgttctcgcctcggcttctcccctcgccatcc<br>acaaaaaatgtgacagcagcagctcccccgcagactccacctggggtgtgcccactcccaccgcgtctc<br>tctccgctaccttaatcgccactcctcccaccggctctctttccgcagttcctacctctggtgttggcatcggc<br>gtcggtgtcgccctacctggattcgagtctggcagctccgctcctggcggttccctgcctggactctcccc<br>ccccgtgggcgtgctccctgttggcgccctccctggcatcgctgcgcctggcgtgcccactggcgtcgcc<br>ctcggcgtcacactccccatactcggttctggcagctcctccgctcctggcagctccttgcctggagtctcc<br>ctccctggtggctccctccccggtagcttgcctggagtccccctccctggtagctccacgcctggcagttcc<br>gcccctggtggcatcgatgtcggtgtcgccctccctggtgcctccgcccccgttggctctattcctggcag<br>ccccgcccctggcgtgccagctggtgttgccgtcggcgtcaacctccccggactcgagtctggcggctc<br>ctctgcgcctggcagctccgtccctggaatccccgcccctggcatccccgctgcagtccccgctggcgtct

	ccctcaccgtcgccctccccttcggcggcgcctctacccctactccgtcctctcctaccggtcagagcccta<br>cttctcatgcgtccatctcggtatccatccccaccgctgtccccacctattcaatccccgcctggtccgattct<br>gtccccgtcccctcgcccaccgctggttcctcctccccccatcactccggctcatctgatgacggctccaca<br>agccaatgcaacactggttccatccagtgctgcaacaaattcggagacgccagctccgacgacatgag<br>gagcttcctgctaggcatcggcgccccccctcaaaggccttagcggtctcgtcggcgcccagtgctccccg<br>atcgaccccgccggtttgggcaacggcgagtgcaagcaggcgcctgtctgctgcaagaacgacgcac<br>agggtggccttgtcagcattggctgcgtcccgatcacgctctga
Gf.hyd11347	atgtcgtcctctatgacccatatggccgtggacctgccggagggagaactagtcgccgtgcggtcgccg<br>ttggagcacaccgaggacccacccaactcagccagcatgcagttcaagctctccctcatctccgccgtca<br>ccctcgccgttctcgcctccgcctctcccctcgccatccacaaaaaatgtgacagcagcagctcccccgca<br>cactccacctggggcgcgcccacccccaccgcgtctctctccgctaccttaaccgccactcttcccaccgg<br>ctctctttccgcagttcctacctctggtgttggcatcggcgtcagcgtcggcggcctacctggactcgagtc<br>tggcagctccgctcctggcggttccctgcctggactctccgtccccgtgggcttgcctggaaccccccctcg<br>ctggcgtcgccctcggcgtcacactccccatactcggttctggcagctcctccgctcctggcagctccttgc<br>ctggagtctccctccccggtagcttgcctggagtctccctccctggtagctccacgcctggcagttccgcc<br>cctggtggcatcgatgtcggtgtcgccctccctggtgcctccgcccccgttggctctattcctggcagccc<br>cgcccctggcgggcccgctggtgttgccgtcggcgtcaacctccccggactcgagtctgccggctcctct<br>gcccctggcagctccgtccctggaatccccgcccctggcatccccgctgcagtccccgctggcgtctccct<br>caccgtcgccctccccttcggcggcgcctctacccctactccgtcctctcctaccggtcagagccccacttct<br>catgcgtccatttcggtatccatccccaccgctgtccccacctattcaatccccgcctggtccgattctgtcc<br>ccgtcccctcgcccaccgctggttccccccatcactccggctcatctgatgacggctccacaagccaatgc<br>aacactggttccatccagtgctgcaacaaattcggagacgccagctccgacgacatgaggagcttcctg<br>ctaggcatcggcgccccccctcaaaggccttagcggtctcgtcggcgcccagtgctccccgatctga
Gf.hyd12081	atgttctcccgcttctccgccatcttcgtctccaccgttgccctctctgttcttgcgaccgccactcccaactac<br>gaggccaagcgctggagcacgtcctccactgccgaagccaccacgacggtcaccgtgaccgctaccgc<br>ccccgcctcgaccgagagcgcgggcagctgcaacgtcggcgacctccagtgctgcaatagcgttgaat<br>cc
Gf.hyd12082	atgtccgcatatggtgacgtcgttaatttcgaatcggcaccgcttgtcgctgtcctgcggacgcggatcct<br>ggacgcacgcacgagtgcgcggattgcccccgccgacatcacacgaacggagatgcaaatatcacttc<br>tccccgagggcgcgggtcatgcccccagtctttgcaggtatataaaccctcagttcgagctttcaaattcc<br>ccagaccgaccagcagctctcctacacacactcgtcccactcaccatactcacagcaaacatgttctcccg<br>cttctccgctgtcttcgtc<br>gccactgttgccctctctgttcttgcgaccgccaccccggccaagcgctggagcacgaccgcggcgacg<br>accgccaaggtcaccacgactgccaaggtcaccacgacggtcaccgtgaccgctaccgcccccgcctc<br>gaccgagagcgcgggcagctgcaatgttggcgacatccagtgctgcgatagcgtcgaatccgccgact<br>cccctgcagccgttggccttctgggcctgcttggaatcgtcgtagatggcttggacgtgcttctcggcctg<br>aactgctcgccgctcagcattattggcatcggcggtgggtcgtgcgatgcgaacccggtctgctgcgag<br>aataataacgtgggtggtctcatctccatcgggtgtatccccatcatcctctag
Gf.hyd12802	atgttctcccgcttctccgctgtcttcgtcgccaccgttgccctctctgttcttgcgaccgccaccccggcca<br>agcgctggagcacgaccgcggcgacgaccgccaaggtcaccacgactgccaaagtcaccacgacgg<br>tcaccgtgaccgctaccgcccccgcctcgaccgagagcgcgggcagctgcaacgtcggcagcctccag<br>tgctgcgatagcgtcgaatccgccgactcccctgcagccgttggccttctgggcctgcttggaatcgtcgt<br>agatggcttggacgtgcttctcggcctgaactgctcgccgctcagcattattggcatcggcggtgggtca<br>tgtgatgcgaacccggtctgctgcgagaataataacgtgggtggtctcatctccatcgggtgtatcccca

	tcatcctctag
Gf.hyd13942	atgttcggaaatcgtgcactctttgcgacgacgtctctcgcacttcttgcggctgccattccccagtcgacc<br>ccaacgcagtgcgacttcctcacatcgccccttctctgttgccagcaagtcctgtctggcgacttgcctatc<br>ctcggtcccattctgcaatcgcttgggataccgcttctcggcgccggcaccgaagtcgggctgggctgtt<br>cgtccactaattccatcgaggtcctgttgggagcttgcgccaccggagacgtggtctgttgtgacaacaa<br>cgatttcgggggaattatcgcacttggatgccaaaatgttactggtttgctatga
Gf.hyd14947	atgttcgcccgcatcgctgctgtttccttcctcgcccttgcggctgccacctctgctaactcgcagtgcaaca<br>ccggtcccatccagtgctgccagagtgttcagcaggcgaactccgctgctggcaccgctcttctctccatg<br>ttgggcgttgtgctgaatgatccgactgtgttgattggtggccaatgctcccccatctctgccgttggcgtt<br>ggtagcggctcagagtgcaatgcgcaccctgtgtgctgcactaacaacaacgttggtggagtgatttct<br>gttggctgtgttcccgtccagctctaa
Gf.hyd15024	atgttcaagctctcccgtgctctcgtgctcttctgcttcgccctctccctcgccctccttgcggtcgccaggcc<br>cggcgcatcccctcccgactgcaccaccacctccttccctccccccacaaccaccgtcaccgtcaccgccc<br>ccgccccgacccccaccggcaccgaccagtgcaacaccggcaacatccagtgctgcgatagctcctcgt<br>ccccggtgggctcgctcattttgggcctgctcggtatcgtcgtagacctcgtagatggcgtcgacgtgct<br>gctcggattgggctgcaacgcgattgtcgtcgtgggtgtcggcgagagcccccagtgcgcggcgacg<br>cccgtgtgctgcgagaacaacagcgtcggcggcctcatctccatcggctgcatcgtcatcattctctaa
Gf.hyd20923	atgatgcggggacgctctgacgttatccagtccataatgttcgccaaacgtgcactcctcactacaacctc<br>cttcgtgctccttgcgaccgcccagaattgcagcattctgtcgaccaaccttctctgttgcgacgagactca<br>gtctggtggcaccattactgggctcaactgctcgtctaccaagctggcggctatcctgctgggggttgt<br>ctcactggaaatctagcctgctgtgacagtagcggttcggggattggcgggttcgaatgcgaggctcca<br>gccttactaccgtctgcagtattgagcgttttgggttaa
Gf.hyd21629	gccgaagccaccacgacggtcaccgtgaccgctaccgcccccgcctcgaccgagagcgcgggcagct<br>gcaacgtcggcgacctccagtgctgcaatagcgttgaatccgccagctcccctgcagcgactacccttct<br>cggcctgctcggcattgtcgtcgatggcttggatgtgcttctcggcctgggctgctcgccgatcagcgtg<br>atcggcgtcggctccggctcggcgtgcgatgcgtctccggtctgctgcgagaataacaacgtgggcgg<br>tctcatctccatcggatgtgtccccgtcatcctctag
Gf.hyd15802	atgttcaagctctcccgtgtgctcgcgctcttctgcttcgcactctccctcgccctccttgcggtcgccaggc<br>ccggcgcgtcccctccagactgcaccaccaccacctccttccctccccccacaaccaccgtcaccgtcacc<br>gcacccgccccgacccccaccggcaccgaccagcacgtccgccccgcccatcccggctctccactactg<br>acaagttacttttgcgcgcagtcctcgtccccggtgggctcgctcattttgggcctgctcggtatcgtcgta<br>gacctcgtagacggcgtcgacgtgctgctcggattgggctgcaacgcgatcgtcgtcgtcggcgccgg<br>cgagagcccacagtgcgcggcgacgcccgtgtgctgcgagaacaacagtgtgggcggcctcatctcc<br>atcggctgcatcgtcatcattctctaa
Gf.hyd19717	atgttctctcgcttctccgcactcttcgtctccaccgtcgccctctccgttcttgcggtcgccacccccaaccc<br>agtgaagcgcggaggagagcccacgacgacgacggtcaaggccaccacgacgatcaccgtgactgc<br>caccgcgcccgcggcgagcgagagcgcaggttcctgcaacgtcagccccatccagtgctgcgagtcca<br>ccgagatcgcatcgtctgctgcggggactacgctgctgggcctgctcggcatcgtcctcaccgacctcaa<br>cgtgctgctcggcctgaactgctcgcccctcagcataatcggcgtcggctccggctcggcgtgtgatgc<br>gtctccggtctgctgcgagaacaacaatgtg
Gf.hyd4500	atgacatcccttgcacttcttgcggccgccaaccccgctcctgttccccagtcgagtgcagaaggctgcac<br>ccttctcgtcgataatctgctctgctgccaggagtttgagccaggcgatgcgcccatcctcggccccatctt<br>tgaactgcttgggctattacttcccggcgttaacactctcgtcggactgcagtgcacgtctaccaacgccg<br>ttgaagtcgcgttgggaagttgtctcgatggtaatacgtctgctaactccgtttgtgcgcaggaggagtc

	gttgcccttggatgccatcctgtga
gapdh	atgccagtcaaggtcggaatcaacgggtgcgtcggctgtcgcattggccgtattgtgctccgtaatgctc<br>tcctcaaccccgaaatcgaggtcgtcgctgtgaacgatcccttcattgacctcgaatacatggtgtacatg<br>ttcaagtacgactccgtccatggtcgcttcaagggctccatcgaggccaagaatggcaagctctacgttg<br>agggcaagcctatctcagtataccaggagaaggatgccgccaacatcaagtggagcgagactggtgc<br>tgcttacattgtcgagtccaccggtgttttcaccaccactgagaaggcatctgcgcatttgaagggtggtg<br>cgaagaaggtcatcatctctgccccctctgctgatgctcccatgttcgtctgcggtgtcaaccttgacgcct<br>acgactccaagtacactgttatctccaacgcgtcctgcaccaccaactgcttggcgcctcttgccaagatc<br>atcaacgacaacttcggtatcgtcgagggtctcatgactactattcacgccactaccgcgacccagcgca<br>ccgtcgacggtccttctcacaaagactggcgcggtggacgtgccgttggcaacaacatcatcccgtcttc<br>caccggtgccgccaaggccgtcggcaaggtcatccccagcctgaatggcaagctcaccggtctctcgtt<br>ccgtgtgcccaccgttgacgtctccgtggtcgaccttgtcgtgcgcctcgagaagagcgcgagctacga<br>cgagatcaaggctgcggtgaagcgggcgtctgagggcccgatgaagggcatcatgggctacaccga<br>ggagaaggtggtgtcgacagactttactggcaacgacaactcgtcgatctttgacgtggatgcgggcat<br>cgcgctgaacaagaactttgtgaagctgatcgcgtggtacgacaacgagtggggctactcgaagcgc<br>gtgtgcgacctgctcgtgttcgtggcgaagaaggacggcgcgctctga

Table S3. The primers for qRT-PCR of typical hydrophobin* genes used in this study

Primers	Sequence (5′-3′)
P1: *gapdh*F	AGCGAGACTGGTGCTGCTTA
P2: *gapdh*R	CGTTGGAGATAACAGTGTACTTGG
P3: *hgfI*F	CAAGCTCGCCATCTTCGCTAC
P4: *hgfI*R	GTGCGTCGACATCAGAGATG
P5: *hgfII*F	TCGCATCGCTGCCGTTTCCT
P6: *hgfII*R	GACGACGTCCACACTCTGG
P7: *Gf.hyd2041*F	ATTGCTTGGCATTGTCCTGG
P8: *Gf.hyd2041*R	TGCAACCAATGGAAATGAGC
P9: *Gf.hyd6681*F	CATTGTTGGGCTTAACTGTTCG
P10: *Gf.hyd6681*R	AGGGTCGGGTTTTGGCATC
P11: *Gf.hyd6682*F	ACTTGCACTCCTCACTACAAC
P12: G*f.hyd6682*R	GAGCAGTTGAGCCCAGTAAT
P13: *Gf.hyd7622*F	TATTCAGGATTTGAGCGTCCTC
P14: *Gf.hyd7622*R	GTTGTTGTTCGTGCAGCAG
P15: *Gf.hyd8174*F	ACACTGTGACGACCGCAAAC
P16: *Gf.hyd8174*R	CCAAGGACAATGCCCAAGA
P17: *Gf.hyd8531*F	TCTTGCCACCTTCGGATTC
P18: *Gf.hyd8531*R	TCGCACAGCACTGGATG
P19: *Gf.hyd8825*F	CACCGTCGCCTTCTTCTACT
P20: *Gf.hyd8825*R	TTGTTGTTCGTGCAGCAGA
P21: *Gf.hyd9954*F	CCTCAGTTGGCGTCGTTGT

P22: *Gf.hyd9954*R	TGATTGAAGGTTGACCGGAAT
P23: *Gf.hyd10182*F	TGCTGAATGATCCGACTGTG
P24: *Gf.hyd10182*R	AGAAAGCACTCCACCAACAT
P25: *Gf.hyd11240*F	AGGGTGGCCTTGTCAGCATT
P26:*Gf.hyd11240*R	TTGTGGAGCCGTCATCAGA
P27:*Gf.hyd11347*F	CACTTCTCATGCGTCCATTTC
P28:*Gf.hyd11347*R	TTGTGGAGCCGTCATCAGA
P29: *Gf.hyd12081*F	CTTCTCCGCCATCTTCGTCTC
P30: *Gf.hyd12081*R	GCTATTGCAGCACTGGAGGT
P31: *Gf.hyd12082*F	TCGTTAATTTCGAATCGGCAC
P32: *Gf.hyd12082*R	GAGTGTGTGTAGGAGAGCTGCTG
P33: *Gf.hyd12802*F	ATGGCTTGGACGTGCTTCT
P34: *Gf.hyd12802*R	GGATACACCCGATGGAGATG
P35: *Gf.hyd13942*F	TCTGGCGACTTGCCTATCCT
P36: *Gf.hyd13942*R	TGGAATTAGTGGACGAACAGC
P37: *Gf.hyd14947*F	TTGTGCTGAATGATCCGACT
P38: *Gf.hyd14947*R	CAGCCAACAGAAATCACTCCA
P39: *Gf.hyd15024*F	GTGCTCTCGTGCTCTTCTGCT
P40: *Gf.hyd15024*R	GCTATCGCAGCACTGGATGTT
P41: *Gf.hyd20923*F	TCCTCACTACAACCTCCTTCGT
P42: *Gf.hyd20923*R	TCACAGCAGGCTAGATTTCCA
P43: *Gf.hyd21629*F	TTGTCGTCGATGGCTTGG
P44: *Gf.hyd21629*R	TGTTATTCTCGCAGCAGACC

Typical hydrophobin* represents the hydrophobin contain eight conserved cysteine residues.

References

1. Lim, H. J.; Lee, E. H.; Yoon, Y.; Chua, B.; Son, A., Portable lysis apparatus for rapid single-step DNA extraction of Bacillus subtilis. *J Appl Microbiol* **2016,** *120* (2), 379-87.

2. Bankevich, A.; Nurk, S.; Antipov, D.; Gurevich, A. A.; Dvorkin, M.; Kulikov, A. S.; Lesin, V. M.; Nikolenko, S. I.; Pham, S.; Prjibelski, A. D.; Pyshkin, A. V.; Sirotkin, A. V.; Vyahhi, N.; Tesler, G.; Alekseyev, M. A.; Pevzner, P. A., SPAdes: a new genome assembly algorithm and its applications to single-cell sequencing. *J Comput Biol* **2012,** *19* (5), 455-77.

3. Seemann, T., Prokka: rapid prokaryotic genome annotation. *Bioinformatics* **2014,** *30* (14), 2068-9.

4. Chan, P. P.; Lowe, T. M., tRNAscan-SE: Searching for tRNA Genes in Genomic Sequences. *Methods Mol Biol* **2019,** *1962*, 1-14.

5. Lagesen, K.; Hallin, P.; Rødland, E. A.; Staerfeldt, H. H.; Rognes, T.; Ussery, D. W., RNAmmer: consistent and rapid annotation of ribosomal RNA genes. *Nucleic Acids Res* **2007,** *35* (9), 3100-8.

6. Birney, E.; Clamp, M.; Durbin, R., GeneWise and Genomewise., *Genome Research* **2004,** *14* (5), 988-995.

7. Ma, Z.; Song, B.; Yu, L.; Yang, J.; Han, Z.; Yang, J.; Wang, B.; Song, D.; Xu, H.; Qiao, M., Efficient expression of hydrophobin HGFII-his via *POT1*-mediated δ integration strategy and its potential in curcumin nanoformulation. *Colloids and Surfaces A: Physicochemical and Engineering Aspects* **2023**, *656*.

8. Cittadino, GM.; Andrews, J.; Purewal, H.; Estanislao, Acuña, Avila, P.; Arnone, JT., Functional Clustering of Metabolically Related Genes Is Conserved across Dikarya. *J Fungi* (Basel). **2023**, 28;9(5):*523*.
